# NAD^+^ prevents chronic kidney disease by activating renal tubular metabolism

**DOI:** 10.1172/jci.insight.181443

**Published:** 2025-03-10

**Authors:** Bryce A. Jones, Debora L. Gisch, Komuraiah Myakala, Amber Sadiq, Ying-Hua Cheng, Elizaveta Taranenko, Julia Panov, Kyle Korolowicz, Ricardo Melo Ferreira, Xiaoping Yang, Briana A. Santo, Katherine C. Allen, Teruhiko Yoshida, Xiaoxin X. Wang, Avi Z. Rosenberg, Sanjay Jain, Michael T. Eadon, Moshe Levi

**Affiliations:** 1 Department of Pharmacology and Physiology, Georgetown University, Washington, DC, USA.; 2 Department of Medicine, Division of Nephrology, Indiana University School of Medicine, Indianapolis, Indiana, USA.; 3 Department of Biochemistry and Molecular & Cellular Biology, Georgetown University, Washington, DC, USA.; 4 Department of Biology, University of La Verne, La Verne, California, USA.; 5 Tauber Bioinformatics Research Center, University of Haifa, Haifa, Israel.; 6 Department of Microbiology and Immunology, Georgetown University, Washington, DC, USA.; 7 Department of Pathology, Johns Hopkins University School of Medicine, Baltimore, Maryland, USA.; 8 National Institute of Diabetes and Digestive and Kidney Diseases, National Institutes of Health, Bethesda, Maryland, USA.; 9 Department of Medicine, Washington University School of Medicine, St. Louis, Missouri, USA.; 10 Center for Biological and Biomedical Engineering, Georgetown University, Washington, DC, USA.

**Keywords:** Nephrology, Chronic kidney disease, Molecular biology, Pharmacology

## Abstract

Chronic kidney disease (CKD) is associated with renal metabolic disturbances, including impaired fatty acid oxidation (FAO). Nicotinamide adenine dinucleotide (NAD^+^) is a small molecule that participates in hundreds of metabolism-related reactions. NAD^+^ levels are decreased in CKD, and NAD^+^ supplementation is protective. However, both the mechanism of how NAD^+^ supplementation protects from CKD, as well as the cell types involved, are poorly understood. Using a mouse model of Alport syndrome, we show that nicotinamide riboside (NR), an NAD^+^ precursor, stimulated renal PPARα signaling and restored FAO in the proximal tubules, thereby protecting from CKD in both sexes. Bulk RNA-sequencing showed that renal metabolic pathways were impaired in Alport mice and activated by NR in both sexes. These transcriptional changes were confirmed by orthogonal imaging techniques and biochemical assays. Single-nuclei RNA sequencing and spatial transcriptomics, both the first of their kind to our knowledge from Alport mice, showed that NAD^+^ supplementation restored FAO in proximal tubule cells. Finally, we also report, for the first time to our knowledge, sex differences at the transcriptional level in this Alport model. In summary, the data herein identify a nephroprotective mechanism of NAD^+^ supplementation in CKD, and they demonstrate that this benefit localizes to the proximal tubule cells.

## Abstract 2

NAD^+^ supplementation might protect from chronic kidney disease. Our work identifies a mechanism and location responsible for this potential benefit.

## Introduction

Chronic kidney disease (CKD) is a clinical diagnosis characterized by the gradual loss of renal function. The pathophysiology of CKD is complex, and kidney diseases of distinct etiologies can all converge on CKD (1). Nevertheless, several unifying mechanisms emerge when comparing healthy kidneys with their diseased counterparts. These include the progressive worsening of renal fibrosis, inflammation, and metabolic disturbances. This suggests that treatments that prevent these changes might also prevent the associated loss of renal function. Herein, we focus on preventing the progression of kidney disease by activating renal metabolism with the nicotinamide adenine dinucleotide (NAD^+^) precursor, nicotinamide riboside (NR).

Decreased NAD^+^ levels contribute to acute kidney injury (AKI), and NAD^+^ supplementation is protective in models of AKI (2–4). Using both gain- and loss-of-function transgenic mice, Tran et al. (5) comprehensively showed that the PPARγ coactivator 1-α (PGC-1α) protects from AKI by upregulating genes in the de novo NAD^+^ synthesis pathway in the renal tubules, and these effects are replicated by NAD^+^ supplementation. PGC-1α is a coactivator that controls metabolism-related gene regulatory networks, including by direct interaction with PPARα (6). Their data clearly indicate that improvements in renal tubular mitochondrial function, including fatty acid oxidation (FAO), substantially contribute to the protective effects of NAD^+^ supplementation in AKI (5).

Recent studies suggest that reduced NAD^+^ may also play a role in CKD. Endogenous NAD^+^ biosynthesis is impaired in CKD, and there is a corresponding decrease in levels of NAD^+^ and its related metabolites (7, 8). Consistent with this, both promoting NAD^+^ salvage and preventing NAD^+^ breakdown protect from CKD (9, 10). Furthermore, pharmacological NAD^+^ supplementation has generally been shown to protect against CKD (11–16). However, unlike in AKI, the mechanism of how NAD^+^ supplementation protects the kidney in CKD is still poorly understood. A key limitation is that no study thus far has investigated the cell type–specific effects of exogenous NAD^+^ supplementation in a model of CKD, which may affect the podocytes or the renal tubules to varying extents.

In a recent study, we demonstrated that NR treatment of diabetic mice restored mitochondrial function, including sirtuin 3 activation, thereby preventing mitochondrial damage. This reduced expression of the cyclic GMP-AMP synthase/stimulator of interferon genes pathway and thus protected the kidney. Importantly, we also identified changes in metabolic genes, including NR-mediated increases in mRNA transcripts for PGC-1α (*Ppargc1a*), nuclear respiratory factor 1 (*Nrf1*), mitochondrial transcription factor A (*Tfam*), carnitine palmitoyltransferase 1-α (*Cpt1a*), medium-chain acyl-coenzyme A dehydrogenase (MCAD; *Acadm*), and long-chain acyl-coenzyme A dehydrogenase (*Acadl*) (15). These genes are encoded in the nuclear genome, and thus, it is unlikely that the changes were a direct result of mitochondrial sirtuin 3. Instead, it implies a mechanism of NAD^+^ supplementation that acts at the transcriptional level to modulate expression of key metabolic genes. We therefore sought to identify the molecular mechanism underlying this change.

To begin to dissect this mechanism, as well as cell types responsible, we returned to the NAD^+^ supplementation data reported from models of AKI. We found the data reported by Tran et al. (5) very compelling evidence for the role of PGC-1α in the renal tubules and consistent with the transcriptional activation of metabolic genes that we observed in our recent study (15). Furthermore, mitochondrial dysfunction and associated FAO defects are key metabolic disturbances that drive CKD (17, 18), and restoring renal metabolism has been shown to be protective (19). Given this rationale, we hypothesized that NAD^+^ supplementation with NR protects from CKD by activating renal metabolism in the proximal tubules.

We tested this hypothesis with a 3-step sequential approach. We first tested NR in a mouse model of Alport syndrome to show that NAD^+^ supplementation protects against CKD at multiple time points. We then employed biochemical techniques, including bulk RNA sequencing (RNA-Seq) and immunoblotting, to assess metabolic dysregulation. Finally, we performed single-nuclei RNA-Seq (snRNA-Seq) and spatial transcriptomics (ST) to show that NAD^+^ supplementation enhances renal metabolism in the proximal tubules. The results presented herein provide strong evidence that NR activates the NAD^+^/PGC-1α/PPARα/FAO axis in the proximal tubules, thereby stimulating metabolism and protecting the kidney.

## Results

### Alport mice have reduced NAD^+^ levels and impaired renal metabolism.

**Figure 1:**
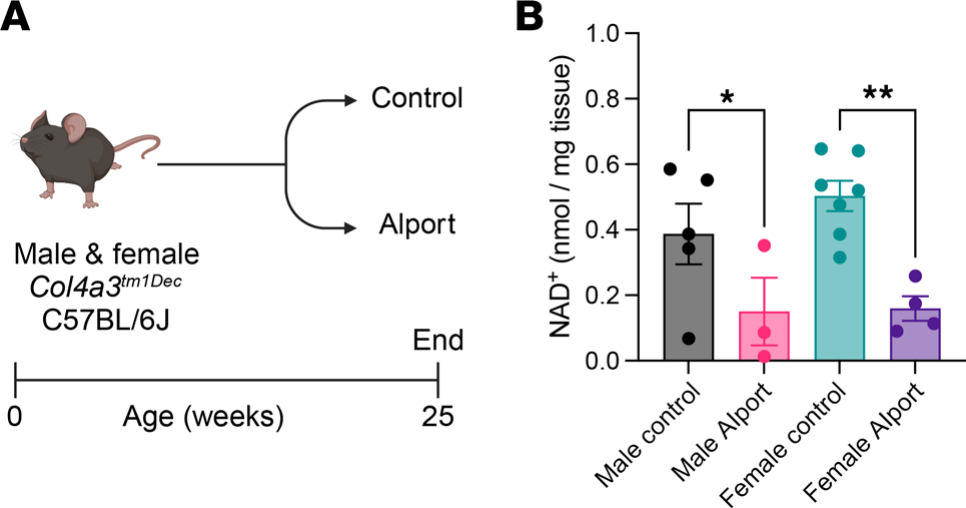
Kidney NAD^+^ is reduced in Alport mice. (**A**) Experimental design: Control and Alport mice of both sexes were sacrificed at 25 weeks of age. (**B**) Alport mice had lower levels of kidney NAD^+^ than control mice. Significance was determined by 1-way ANOVA with the Holm-Šídák correction for multiple comparisons. *P < 0.05, **P < 0.01. Data are expressed as the means ± SEM, and each datum represents 1 mouse.

To verify that pathways related to both NAD^+^ and renal metabolism are dysregulated in Alport mice, we reanalyzed previously published RNA-Seq data from 2 independent experiments (20, 21). Gene ontology (GO) enrichment and Kyoto Encyclopedia of Genes and Genomes (KEGG) pathway analyses were performed on the 500 most downregulated genes in Alport mice, and both NAD^+^ biosynthetic pathways (Supplemental Figure 1, A and B; supplemental material available online with this article; https://doi.org/10.1172/jci.insight.181443DS1) and fatty acid metabolic pathways (Supplemental Figure 2) were significantly enriched. This is in stark contrast with the pathways identified from analyses of upregulated genes, most of which were related to inflammation and fibrosis (Supplemental Figure 1, C and D) (22). Finally, we verified that kidney NAD^+^ levels were lower in male and female Alport mice compared with control mice (Figure 1).

### NAD^+^ supplementation protects Alport mice from kidney disease.

Given that kidney NAD^+^ levels were decreased in Alport mice compared with control mice, we hypothesized that NAD^+^ supplementation with NR would reduce the severity of kidney disease. Our colony of Alport mice on the C57BL/6J background slowly develop kidney disease until death at 35–40 weeks of age, and we investigated 2 time points.

**Figure 2:**
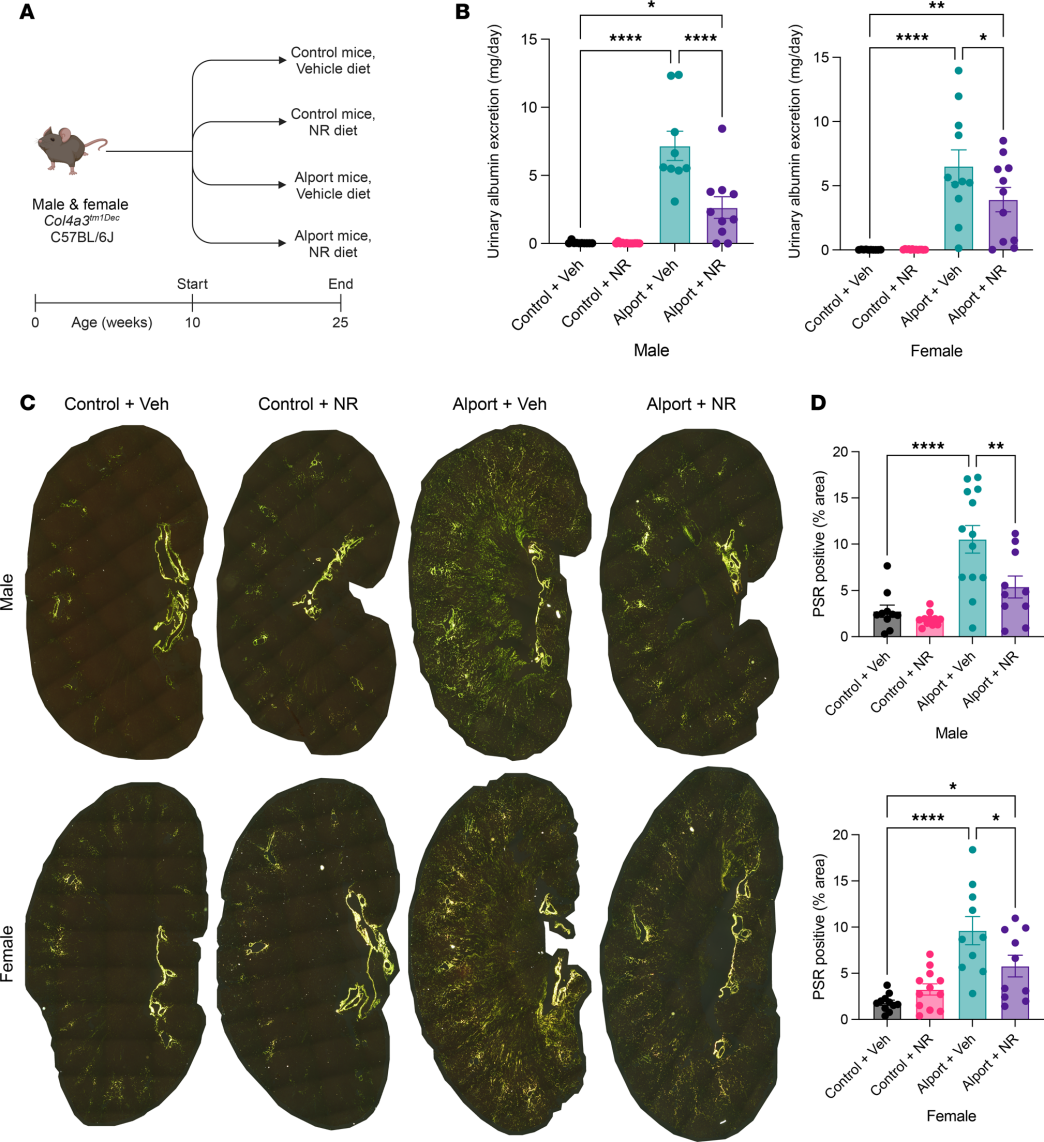
NAD^+^ supplementation protects the kidney in Alport mice. (**A**) Experimental design: Control and Alport mice of both sexes were treated with or without NR between 10 weeks and 25 weeks of age. (**B**) NR treatment reduced 24-hour urinary albumin excretion in Alport mice of both sexes. (**C**) Representative images of PSR-stained kidneys acquired with polarized light. Yellow-green-orange birefringence is highly specific for fibrosis. (**D**) Quantification of PSR-stained kidneys shows that NR treatment reduced renal fibrosis in both sexes. Significance was determined by 1-way ANOVA with the Holm-Šídák correction for multiple comparisons. Data are expressed as the means ± SEM, and each datum represents 1 mouse. *P < 0.05, **P < 0.01, ****P < 0.0001. NAD^+^, nicotinamide adenine dinucleotide; NR, nicotinamide riboside; PSR, Picrosirius red; Veh, vehicle.

In our initial experiment, mice were treated with or without NR between 10 and 25 weeks of age (Figure 2A). We did not observe any changes in echocardiography or blood pressure measurements between the groups, excluding these as potential confounding variables (Supplemental Tables 1 and 2). Twenty-four–hour urinary albumin excretion, a marker of kidney damage, was up to 1,000-fold increased in Alport mice, and NR treatment prevented this increase in both sexes (Figure 2B). Plasma creatinine was unchanged between control and Alport mice at the 25-week time point, consistent with the slowly progressing phenotype of Alport mice on the B6 background (Supplemental Table 3). As shown by polarized microscopy of kidney sections stained with Picrosirius red (PSR), a technique that is highly specific for collagen (23), NR treatment prevented the progression of overall renal fibrosis in both sexes (Figure 2, C and D). In addition, renal cortical tubulointerstitial fibrosis — quantified by excluding medullary, vascular bundle, and glomerular contributions — was increased in Alport mice and reduced by NR treatment in both sexes (Supplemental Figure 3, A and B). Renal inflammatory infiltrate was also increased in Alport mice and reduced by NR treatment (Supplemental Figure 3, C and D). Finally, the prevention of renal fibrosis was secondarily verified by immunoblotting for fibronectin, which was reduced in NR-treated male (significant, *P* < 0.001) and female (trend, *P* < 0.10) Alport mice (Supplemental Figure 4).

We then repeated the experiment in both male and female mice, though we aged the mice longer to 35 weeks of age (Supplemental Figure 5A). Twenty-four–hour urinary albumin excretion was increased in Alport mice, and NR treatment ameliorated this increase in both sexes (Supplemental Figure 5B). At this later time point, plasma creatinine was greatly increased in Alport mice compared with control mice, and NR treatment prevented this increase in both sexes (Supplemental Figure 5C). Both our initial and replication experiments had substantial numbers of littermate-matched mice in each group, and they were temporally separated by greater than 1 year.

### NAD^+^ supplementation protects from glomerular and tubular injury in Alport mice.

**Figure 3:**
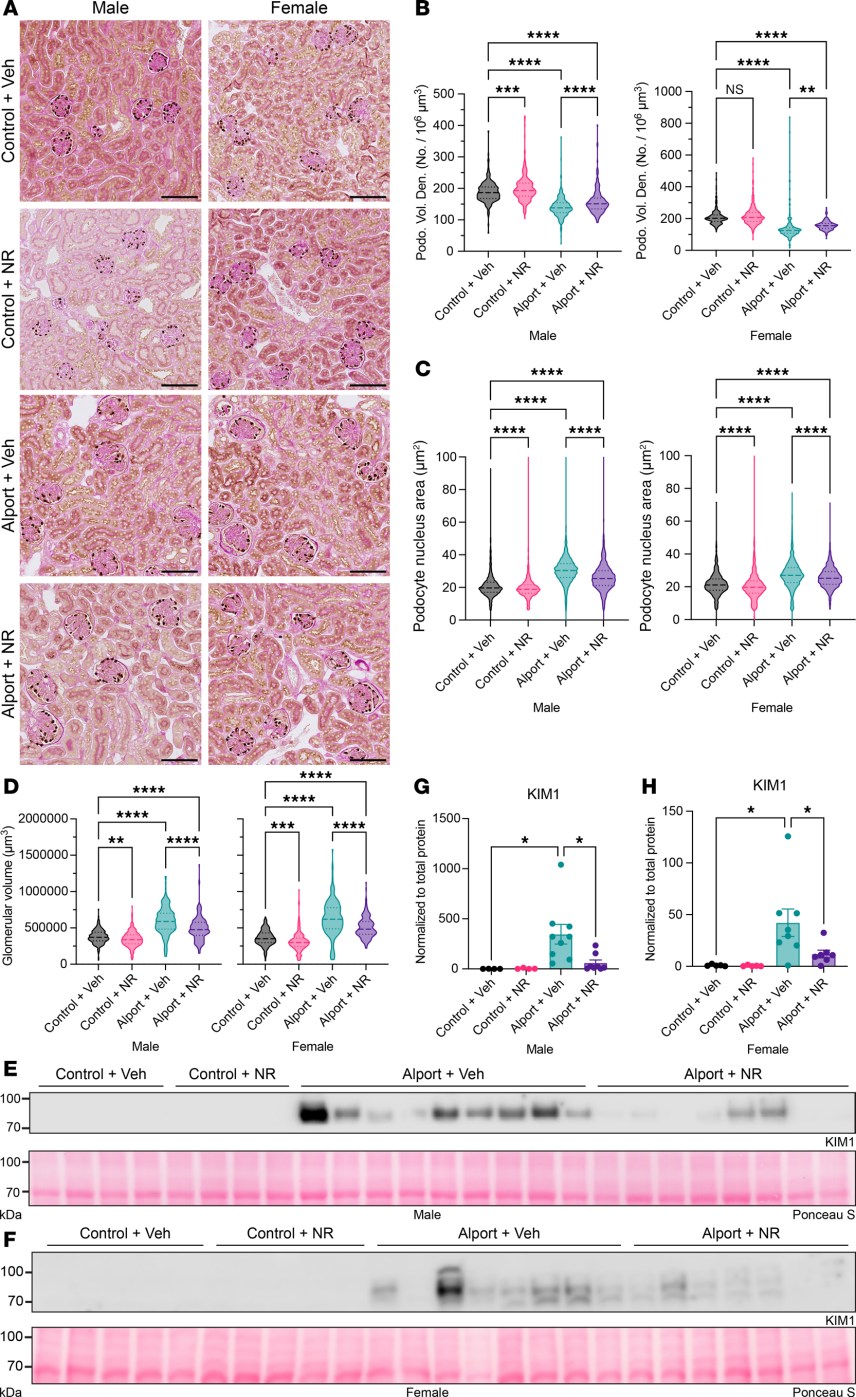
NAD^+^ supplementation prevents both glomerular and tubular injury in Alport mice. (**A**) Representative images of immunohistochemistry for p57^kip2^, followed by periodic acid–Schiff poststaining without hematoxylin counterstaining. Podocyte nuclei are stained brown. (**B**–**D**) Quantification of p57^kip2^ immunostaining with PodoCount, a validated algorithm to analyze p57^kip2^-stained whole-slide images. Podocyte volumetric density (**B**) was reduced in Alport mice and restored by NR treatment in both sexes. Alport mice had podocyte nuclear hypertrophy (**C**) and glomerular hypertrophy (**D**) that was reduced by NR treatment in both sexes. (**E** and **F**) Immunoblots for KIM-1, a tubular injury marker, in male (**E**) and female (**F**) kidney homogenate. Ponceau S, a nonspecific protein stain, was used as a loading control. (**G** and **H**) Quantification of immunoblots (**E** and **F**) shows that KIM-1 is increased in Alport mice and reduced by NR treatment in male (**G**) and female (**H**) mice. Scale bars represent 100 μm. Significance was determined by 1-way ANOVA with the Holm-Šídák correction for multiple comparisons. Data are expressed as the means ± SEM. Each datum represents 1 glomerulus (**B**–**D**) or 1 mouse (**G** and **H**). *P < 0.05, **P < 0.01, ***P < 0.001, ****P < 0.0001. KIM-1, kidney injury molecule-1; NAD^+^, nicotinamide adenine dinucleotide; No., number; NR, nicotinamide riboside; Podo. Vol. Den., podocyte volumetric density; PSR, Picrosirius red; Veh, vehicle.

Glomerular damage was further assessed by immunostaining for the podocyte marker p57^kip2^. Volumetric podocyte density is a podometric that controls for the thickness of the histological section, the size of the podocyte nucleus, and the size of the glomerulus (24–26). Compared with control mice, Alport mice had reduced volumetric podocyte density, both podocyte and glomerular hypertrophy, and an increased mesangial index. NR treatment prevented these pathologic changes in both sexes (Figure 3, A–D, and Supplemental Figure 6A). In females, but not males, the corrected podocyte number per glomerulus was reduced in Alport mice and restored with NR treatment (Supplemental Figure 6B). However, unlike the volumetric podocyte density, the corrected podocyte number per glomerulus does not control for glomerular hypertrophy and should be interpreted with caution. These results, in combination with the reduction in urinary albumin excretion, demonstrate that NR treatment protects from glomerular damage in the Alport model of kidney disease.

Although Alport syndrome is classically associated with a specific glomerular defect, glomerular injury can also cause tubular injury (27, 28). The proximal tubules are highly metabolically active, and they may have limited reserve to compensate for severe glomerular disease. This might be further exacerbated by an NAD^+^ deficiency. We therefore hypothesized that Alport mice would exhibit substantial tubular pathology that was reversed by NR treatment. Consistent with our hypothesis, renal kidney injury molecule 1 (KIM-1) expression, a marker of tubular damage (29, 30), was increased in Alport mice, and NR treatment prevented this increase in both sexes (Figure 3, E–H). We then investigated the mechanism underlying this protective effect.

### Bulk RNA-Seq identifies renal cortical metabolic defects in Alport mice that are prevented by NAD^+^ supplementation.

We performed bulk RNA-Seq on isolated kidney cortex to both support our hypothesis that NAD^+^ supplementation normalizes renal metabolism and identify if PGC-1α/PPARα signaling is a molecular mechanism driving this change. Principal component analysis demonstrated separation by genotype (PC1, 42.7% of variance, *P* = 2.75 × 10^–11^), sex (PC2, 11.3% of variance, *P* = 1.16 × 10^–7^), and treatment (PC3, 7.3% of variance, *P* = 2.07 × 10^–3^). In both sexes, NR treatment shifted Alport samples toward the control genotype, suggesting the prevention of the disease process (Supplemental Figure 7).

For each sex, we then performed GO biological process, KEGG pathway, and transcription factor enrichment analyses (31–33) comparing (a) NR-treated control mice versus vehicle-treated control mice, (b) vehicle-treated Alport mice versus vehicle-treated control mice, and (c) NR-treated Alport mice versus vehicle-treated Alport mice (Supplemental Tables 4–7). The decision to compare these groups was made a priori because they are the most relevant biological comparisons.

**Figure 4:**
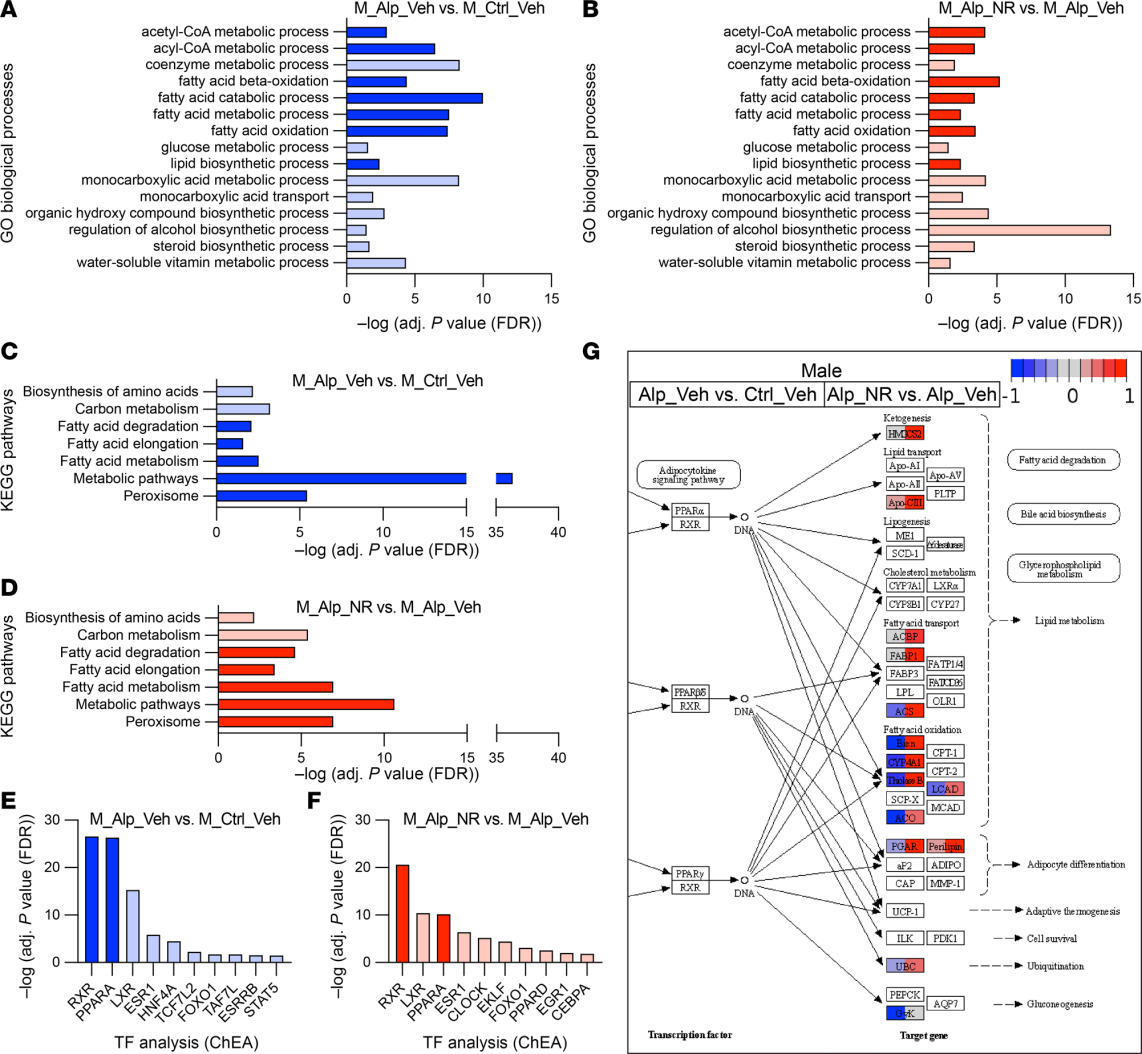
NAD^+^ supplementation activates renal metabolism in male Alport mice. Bulk kidney cortex RNA-Seq data from control and Alport mice, treated with or without NR, were analyzed (*N* = 4 mice per group). (**A** and **B**) GO biological processes that are both (**A**) reduced in vehicle-treated male Alport mice (vs. vehicle-treated male control mice) and (**B**) increased in NR-treated male Alport mice (vs. vehicle-treated male Alport mice) are shown. (**C** and **D**) KEGG pathways that are both (**C**) reduced in vehicle-treated male Alport mice (vs. vehicle-treated male control mice) and (**D**) increased in NR-treated male Alport mice (vs. vehicle-treated male Alport mice) are shown. (**E** and **F**) Transcription factor analyses suggest that the RXR/PPARα gene regulatory network is (**E**) inhibited in vehicle-treated male Alport mice (vs. vehicle-treated male control mice) and (**F**) activated in NR-treated male Alport mice (vs. vehicle-treated male Alport mice). Processes (**A** and **B**), pathways (**C** and **D**), and transcription factors (**E** and **F**) that are directly involved in fatty acid metabolism are highly enriched in all comparisons, emphasized by either dark blue (decreased) or dark red (increased). (**G**) Partial KEGG graph for the PPAR signaling pathway (KEGG Entry No. 03320). The left and right sides of each gene box represent data from vehicle-treated male Alport mice (vs. vehicle-treated male control mice) and NR-treated male Alport mice (vs. vehicle-treated male Alport mice), respectively. The subpathway that was the most restored by NR treatment was FAO. Alp, Alport; ChEA, ChIP Enrichment Analysis; Ctrl, control; FAO, fatty acid oxidation; GO, gene ontology; KEGG, Kyoto Encyclopedia of Genes and Genomes; M, male; NAD^+^, nicotinamide adenine dinucleotide; NR, nicotinamide riboside; RXR, retinoid X receptor; TF, transcription factor; Veh, vehicle.

Consistent with our hypothesis, GO enrichment analyses revealed that numerous metabolism-related biological processes were changed between the groups. In both sexes, GO biological processes involving fatty acids, including fatty acid β-oxidation, were among the most highly enriched set of processes that were simultaneously downregulated in vehicle-treated Alport mice (vs. vehicle-treated control mice) (Figure 4A and Supplemental Figure 8A) and upregulated in NR-treated Alport mice (vs. vehicle-treated Alport mice) (Figure 4B and Supplemental Figure 8B). Many GO biological processes that are integral to energy metabolism were also simultaneously enriched in both comparisons, including acetyl-CoA and acyl-CoA metabolic processes (Figure 4, A and B, and Supplemental Figure 8, A and B). KEGG pathway analyses yielded similar results in both sexes (Figure 4, C and D, and Supplemental Figure 8, C and D). The severe impairment of renal metabolism in Alport mice was evident by the downregulation of genes involved in metabolic pathways (KEGG Entry No. 01100) with FDR-adjusted *P* value approaching 40 orders of magnitude in both sexes (Figure 4C and Supplemental Figure 8C). Transcription factor analyses revealed differential activity of the retinoid X receptor (RXR) and PPARα heterodimer (RXR/PPARα) as a likely candidate underlying the observed transcriptional differences (Figure 4, E and F, and Supplemental Figure 8, E and F).

Based on the data from the GO enrichment, KEGG pathway, and transcription factor analyses, we plotted the gene changes on KEGG graphs for the PPAR signaling pathway (KEGG Entry No. 03320) (Figure 4G, Supplemental Figure 8G, and Supplemental Figures 9 and 10), the peroxisome (KEGG Entry No. 04146) (Supplemental Figures 11 and 12), and fatty acid degradation (KEGG Entry No. 00071) (Supplemental Figures 13 and 14). A multitude of genes involved in these pathways, especially those related to FAO, were reduced in vehicle-treated Alport mice (compared with vehicle-treated control mice) and restored in NR-treated Alport mice (compared with vehicle-treated Alport mice). Data from both comparisons are visualized simultaneously on the KEGG graphs, the former on the left half of each rectangle and the latter on the right half.

All together, these data represent clear evidence for impaired renal metabolism in Alport mice, including fatty acid metabolism, that is restored by NR treatment. Next, we investigated NR treatment in control mice to verify that NR is directly responsible for normalizing renal metabolism.

### NAD^+^ supplementation activates renal metabolism in control mice, verifying this as a causal mechanism of NR-mediated kidney protection.

Although the dramatic change in metabolic state between vehicle- and NR-treated Alport mice suggests that NR protects the kidney by activating renal metabolism, it is not enough alone to show a causal relationship. In other words, because renal metabolism in Alport mice becomes gradually more impaired as kidney disease progresses, any intervention that reduces kidney disease will also cause a coincidental improvement of renal metabolism. This is not because the intervention activates renal metabolism, per se. It is instead an artifact that arises because the intervention-treated group has less severe kidney disease than the vehicle-treated group at the time of study. To truly determine the mechanism of a drug, it is necessary to investigate its effects in healthy control mice. We therefore compared NR treatment with vehicle treatment in control mice, and we juxtaposed this with the comparison of NR treatment with vehicle treatment in Alport mice.

**Figure 5:**
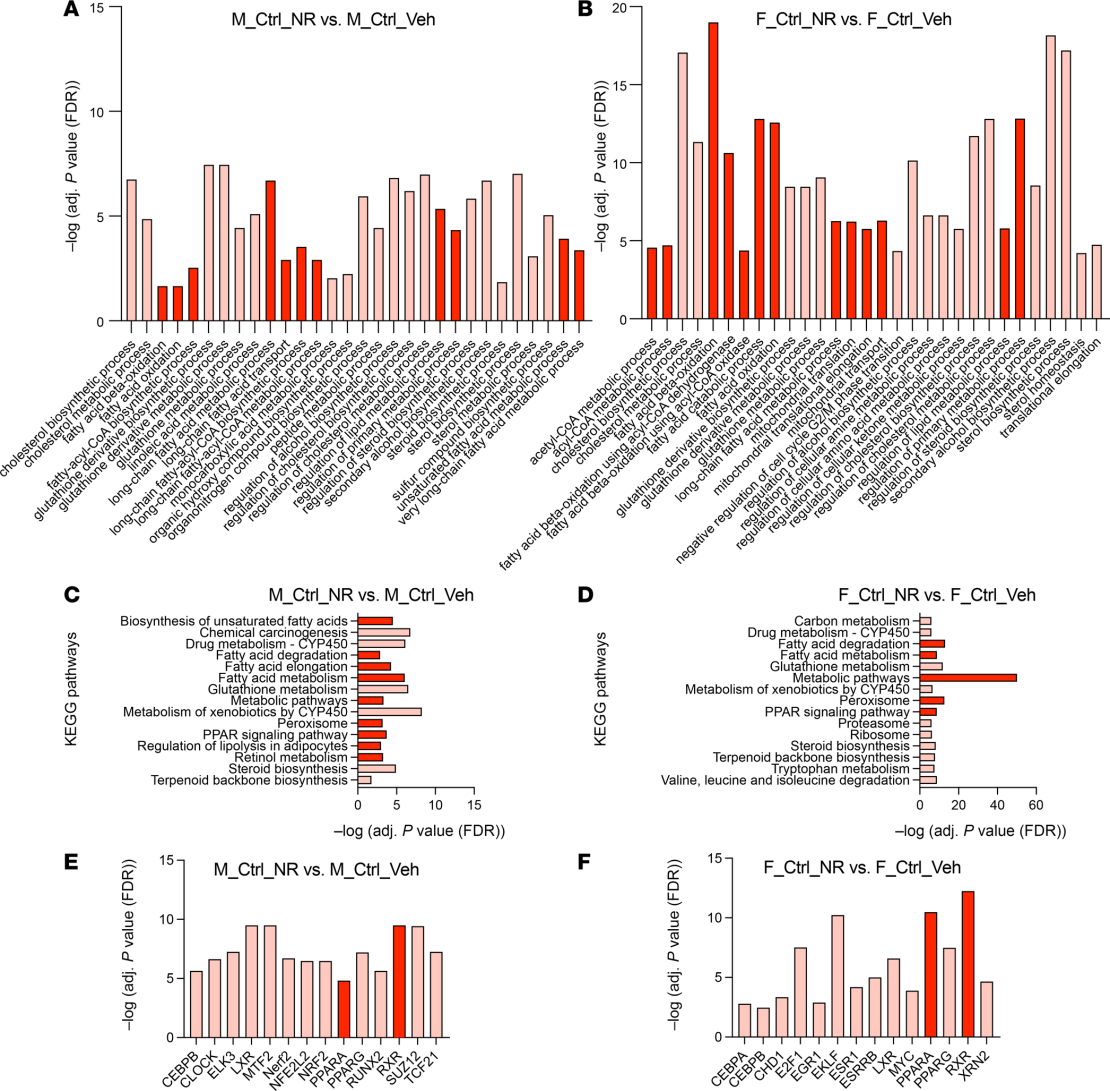
NAD^+^ supplementation activates renal metabolism in control mice, not just in Alport mice. Bulk kidney cortex RNA-Seq data from control and Alport mice, treated with or without NR, were analyzed (*N* = 4 mice per group). GO biological processes and KEGG pathways that were simultaneously upregulated in both NR-treated control mice (vs. vehicle-treated control mice) and NR-treated Alport mice (vs. vehicle-treated Alport mice) were identified. (**A** and **B**) GO biological processes that were increased in NR-treated control mice (vs. vehicle-treated control mice) in males (**A**) and females (**B**). (**C** and **D**) KEGG pathways that were increased in NR-treated control mice (vs. vehicle-treated control mice) in males (**C**) and females (**D**). These data (**A**–**D**) are presented adjacent to the corresponding sex-matched comparisons from NR-treated Alport mice (vs. vehicle-treated Alport mice) in Supplemental Figures 15 and 16. (**E** and **F**) Transcription factor analyses suggest that the RXR/PPARα gene regulatory network is activated in NR-treated control mice (vs. vehicle-treated control mice) in both males (**E**) and females (**F**). Ctrl, control; F, female; GO, gene ontology; KEGG, Kyoto Encyclopedia of Genes and Genomes; M, male; NAD^+^, nicotinamide adenine dinucleotide; NR, nicotinamide riboside; RXR, retinoid X receptor; Veh, vehicle.

In both sexes, metabolism-related GO biological processes were among the most highly enriched set that were simultaneously upregulated in both NR-treated control mice (vs. vehicle-treated control mice) (Figure 5, A and B) and NR-treated Alport mice (vs. vehicle-treated Alport mice) (Supplemental Figure 15). KEGG pathway analyses mirrored these results (Figure 5, C and D, and Supplemental Figure 16). Transcription factor analyses also predicted the activation of RXR/PPARα in the NR-treated control mice (Figure 5, E and F). The striking similarity of NR treatment in both control and Alport mice is demonstrated by visualization on KEGG graphs for the PPAR signaling pathway (KEGG Entry No. 03320) (Supplemental Figures 17 and 18), the peroxisome (KEGG Entry No. 04146) (Supplemental Figures 19 and 20), and fatty acid degradation (KEGG Entry No. 00071) (Supplemental Figures 21 and 22).

In summary, the effect of NR treatment on metabolism-related pathways in control mice was essentially identical to that in Alport mice. These results strongly suggest that NR protects the kidney via activating renal metabolism, more specifically, the RXR/PPARα signaling pathway that stimulates FAO. We then sought to verify these results with orthogonal biochemical assays, but prior to doing so, we investigated the effects of genetic background and sex.

### The renal transcriptome of Alport mice is similar across genetic backgrounds.

Experimental investigations on Alport syndrome typically use collagen, type IV, alpha 3–null (*Col4a3*-null) mice on the 129 or B6 genetic backgrounds and occasionally F1 hybrids thereof. Alport mice on the 129 background develop kidney disease much more rapidly than their counterparts on the B6 background, and 129.B6F1 hybrids have an intermediate phenotype (34). We performed a meta-analysis to verify that the changes in renal transcriptome we observed in our B6 Alport colony are representative of *Col4a3*-null mice on the other backgrounds.

Previously published RNA-Seq data from male control and Alport mice on the 129 and 129.B6F1 backgrounds were compared with vehicle-treated male control and Alport mice from the current study (B6 background). Principal component analysis demonstrated separation by genotype (PC1, 73.2% of variance). The second-largest principal component accounted for only 3.2% of variance, and it was not associated with genetic background (Supplemental Figure 23A). KEGG pathway analyses showed similar impairments in metabolic processes across all genetic backgrounds and the combined meta-analysis (Supplemental Figure 23, B–E).

These data show that the changes in renal transcriptome in Alport mice are independent of genetic background when compared with their respective controls. These results also suggest, but do not prove, that NR treatment would have a similar mechanism of action in all Alport mice regardless of genetic background.

### Male and female Alport mice have distinct inflammatory and fibrotic responses.

Alport syndrome affects both males and females, but it has not yet been reported if the disease progression differs between the sexes at the molecular level. Of the 6 RNA-Seq datasets from Alport mouse kidneys that are deposited in the NCBI Gene Expression Omnibus (GEO), only 1 is from both sexes (20, 21, 35–39). However, it used a unique outbred model that is the first of its kind (36). To address this gap, we analyzed the subset of 16 vehicle-treated mouse kidneys, 4 from each sex/genotype combination.

Principal component analysis showed that the data were tightly clustered with respect to both genotype (PC1, 57.1% of variance, *P* = 4.66 × 10^–8^) and sex (PC2, 14.3% of variance, *P* = 9.27 × 10^–8^) (Supplemental Figure 24A). A total of 114 genes were differentially regulated with both sex and disease, as identified by a sex-genotype interaction (Supplemental Figure 24B). GO enrichment analysis revealed that extracellular matrix and neovascularization processes were comparatively less upregulated in female Alport mice than in male Alport mice when compared with their sex-matched controls (Supplemental Figure 24C). In many cases, genes that were increased in female Alport mice (vs. female control mice), such as the pro-fibrotic and pro-inflammatory genes *C3*, *Col1a2*, *Fgfbp1*, and *Ticam2* (Supplemental Figure 24D), were increased to a greater degree in male Alport mice (vs. male control mice). We refer to this gene set as having a negative sex-genotype interaction because the increase in Alport mice compared with control mice is smaller (or even decreased, not increased) in female mice compared with male mice. An additional 70 genes had a negative sex-genotype interaction (Supplemental Figure 25A), while 40 genes had a positive sex-genotype interaction (Supplemental Figure 25B). To further parse out a potential role of sex as a biological variable, pathway analysis using a modified gene set enrichment analysis algorithm was performed on the entire dataset as previously described (40, 41), not just the subset of genes with a significant sex-genotype interaction. This also identified inflammation-related pathways as differentially regulated with both sex and disease (Supplemental Figure 26). Despite these transcriptional differences, there were no differences between the sexes in histological markers of fibrosis or inflammation (Supplemental Figure 3).

In summary, these data support moderate differential regulation of fibrosis- and inflammation-related pathways between the sexes at the transcriptional level in this mouse model of Alport syndrome. Importantly, neither analysis identified a sex-genotype interaction in metabolism-related pathways, our primary area of interest in this study. Therefore, we chose to study only male mice for all remaining biochemical assays because they seemed to have a more severe phenotype at the transcriptional level. However, the sexes were pooled for the snRNA-Seq experiment because of the outsized benefit these data could have for the scientific community.

### NAD^+^ supplementation restores kidney mitochondrial FAO.

Once we validated the absence of a sex-genotype interaction in metabolism-related pathways, we then returned to investigating the effects of NR treatment. Mitochondrial dysfunction is a well-accepted mechanism seen in models of CKD, including in a mouse model of Alport syndrome (21). In addition, enhancing de novo NAD^+^ synthesis has been shown to improve mitochondrial FAO (42). We therefore hypothesized that mitochondrial FAO would be impaired in Alport mice and restored by NR treatment. To test this, we immunoblotted for key proteins in the mitochondrial FAO pathway: PGC-1α, CPT1α, and MCAD.

**Figure 6:**
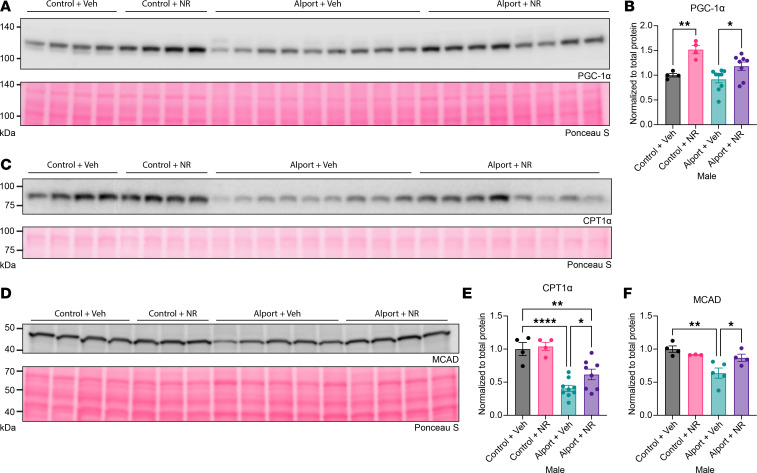
NAD^+^ supplementation activates renal fatty acid metabolism. (**A** and **B**) NR treatment increased kidney PGC-1α in both control and Alport mice. (**C**–**F**) Kidney CPT1α (**C** and **E**) and MCAD (**D** and **F**), key players in mitochondrial FAO, were reduced in Alport mice and restored by NR treatment. Ponceau S, a nonspecific protein stain, was used as a loading control. Significance was determined by 1-way ANOVA with the Holm-Šídák correction for multiple comparisons. Data are expressed as the means ± SEM. Each datum represents 1 mouse. *P < 0.05, **P < 0.01, ****P < 0.0001. CPT1α, carnitine palmitoyltransferase 1-α; FAO, fatty acid oxidation; MCAD, medium-chain acyl-coenzyme A dehydrogenase; NAD^+^, nicotinamide adenine dinucleotide; NR, nicotinamide riboside; PGC-1α, PPARγ coactivator 1-α; Veh, vehicle.

PGC-1α is a coactivator that regulates PPARα function, and both CPT1α (*Cpt1a*) and MCAD (*Acadm*) are PPARα target genes (6, 43, 44). Consistent with the observed effects of NR in both control and Alport mice on RNA-Seq, PGC-1α levels were increased with NR treatment in both genotypes (Figure 6, A and B), and this serves as a good positive control for the effects of NR.

CPT1α and MCAD are both integral to mitochondrial FAO, and defects in either protein can cause severe clinical diseases (45). CPT1α controls fatty acyl-CoA transport into the mitochondria, and it is the rate-limiting step of long-chain FAO and medium-chain FAO of 9 or more carbons in length (46). In our samples, CPT1α was reduced in Alport mice, and it was restored with NR treatment (Figure 6, C and E). Medium-chain fatty acids, at least those up to 8 carbons in length, can enter the mitochondria independent of CPT1α and then are converted to fatty acyl-CoAs once inside (46). However, regardless of how they enter the mitochondria, MCAD catalyzes the first step in mitochondrial FAO of all lengths of medium chain acyl-CoAs (45). In our samples, MCAD was also reduced in Alport mice (vs. control mice) and restored by NR treatment (Figure 6, D and F).

These changes in the key enzymes regulating both medium- and long-chain FAO are robust orthogonal data that validate the transcriptomic changes seen on RNA-Seq. They further demonstrate that Alport mice have impaired fatty acid utilization and that NR protects the kidney by activating renal metabolism.

### NAD^+^ supplementation restores the canonical functions of the proximal tubule, prevents immune cell infiltration, and reduces myofibroblast appearance.

The data presented thus far strongly establish that NR protects the kidney via activating renal cortical transcription of metabolic genes, and this is associated with corresponding improvements in renal expression of mitochondrial FAO proteins. However, they provide little insight into the molecular changes occurring within individual cells. To investigate the cell type–specific effects of NR treatment in Alport syndrome, as well as to show unambiguously that NR stimulates metabolism in the proximal tubules, we performed snRNA-Seq on our samples.

**Figure 7:**
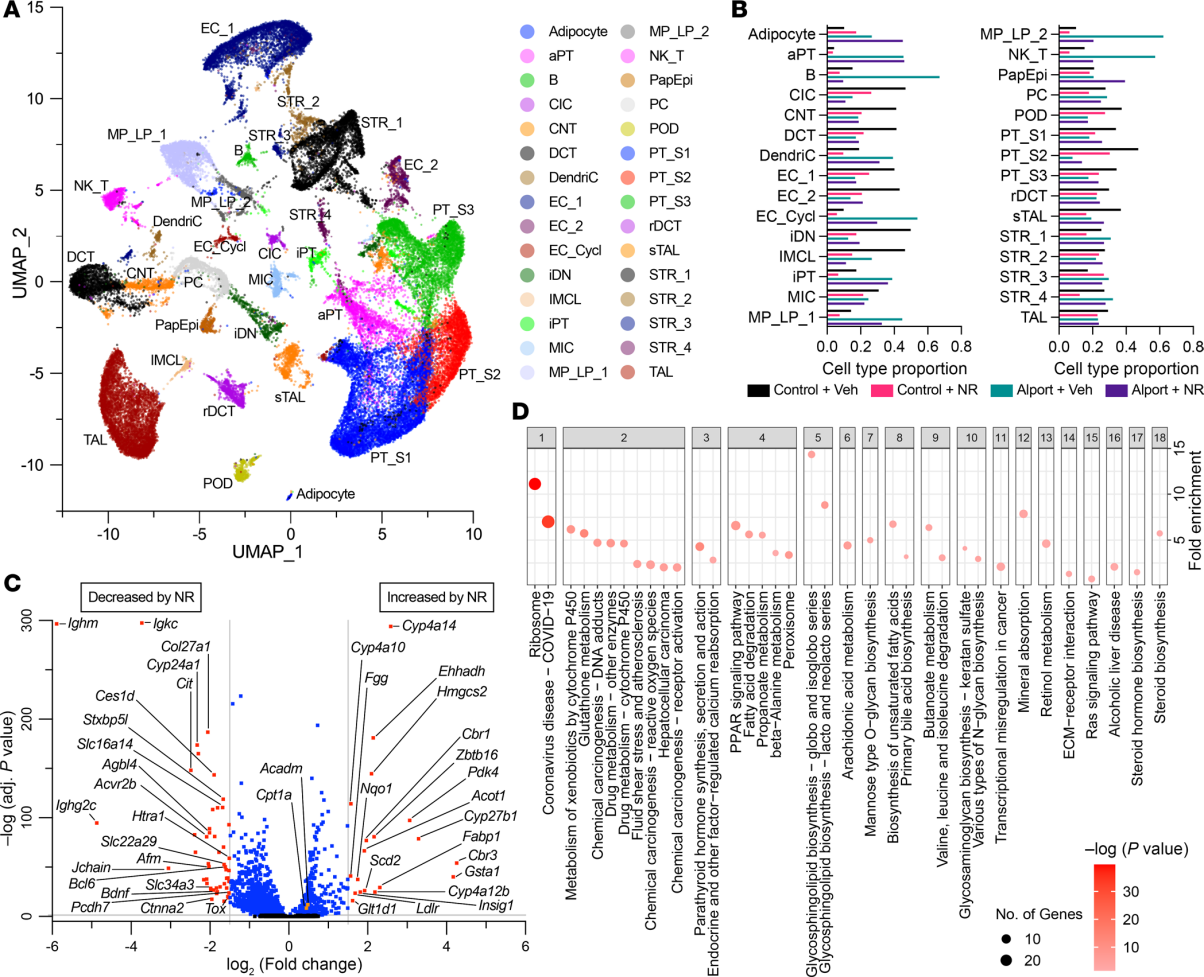
snRNA-Seq of control and Alport mice, with and without NAD^+^ supplementation. (**A**) The snRNA-Seq reduction by UMAP of 49,488 nuclei in 30 clusters. (**B**) Proportion of nuclei per cluster by condition. Myofibroblasts and immune cells were upregulated in Alport mice. The effect was mitigated by NR treatment. (**C**) Differentially expressed genes in proximal tubule cells (PT_S1/S2) of NR-treated Alport mice (vs. vehicle-treated Alport mice). Mice treated with NR had reduced expression of immune signaling transcripts and increased expression of metabolism-related genes (e.g., *Acot1*, *Ehhadh*, and *Insig1*). (**D**) Enriched pathways between NR-treated Alport mice and vehicle-treated Alport mice in the proximal tubule cell. NR treatment significantly impacted translation (ribosome), metabolism, endocrine function, and PPAR signaling in the proximal tubule cell. Ctrl, control; NAD^+^, nicotinamide adenine dinucleotide; NR, nicotinamide riboside; UMAP, uniform manifold approximation and projection; Veh, vehicle. aPT, adaptive proximal tubules; B, B cell; CIC, cortical intercalated cells; CNT, connecting tubule; DCT, distal convoluted tubule; DendriC, dendritic cell; EC_1, endothelial cells 1; EC_2, endothelial cells 2; EC_Cycl, endothelial cells cycling; iDN, injury distal nephron; IMCL, intramedullary collecting cells; iPT, injury proximal tubules; MIC, medullary intercalated cells; MP_LP_1, macrophage or lymphocyte cells 1; MP_LP_2, macrophage or lymphocyte cells 2; NK_T, natural killer or T cell; PapEpi, papillary epithelial cells; PC, principal cell; POD, podocyte; PT_S1, proximal tubules segment 1; PT_S2, proximal tubules segment 2 cells; PT_S3, proximal tubules segment 3 cells 1; rDCT, regenerative distal convoluted tubule; sTAL, stressed thick ascending limb; STR_1, stromal cell 1; STR_2, stromal cell 2; STR_3, stromal cell 3; STR_4, stromal cell 4; TAL, thick ascending limb.

Nuclei from control and Alport mice, both with and without NR treatment, were extracted, sequenced with snRNA-Seq, and merged to create an atlas of 30 cell type clusters (Figure 7A). Clusters were defined by known marker gene expression, and most cell types were represented across all 4 conditions (Supplemental Figure 27). However, the proportion of cell type distribution varied across conditions (Figure 7B). Podocytes and reference proximal tubule cells were more represented in control mice, whereas adaptive proximal tubule and injured proximal tubule had greater representation in Alport mice. Vehicle-treated Alport mice had enrichment of characteristic injury pathways in both the proximal tubule (Supplemental Figure 28) and podocytes (Supplemental Figure 29) as compared with vehicle-treated control mice. These pathways included fatty acid degradation, PPAR signaling, cell adhesion, and immune signaling.

The injury phenotypes of the proximal tubule cell and podocyte in Alport mice shared properties. In the NR-treated proximal tubule, we found transcriptomic evidence supporting restoration of several vital cellular functions: translation (ribosome), metabolism, endocrine function, fatty acid degradation, and PPAR signaling (Figure 7, C and D). In the podocyte, the enriched pathways of NR treatment also included PPAR signaling and fatty acid degradation, though fewer genes were differentially expressed because of the smaller sample size (Supplemental Figure 30).

**Figure 8:**
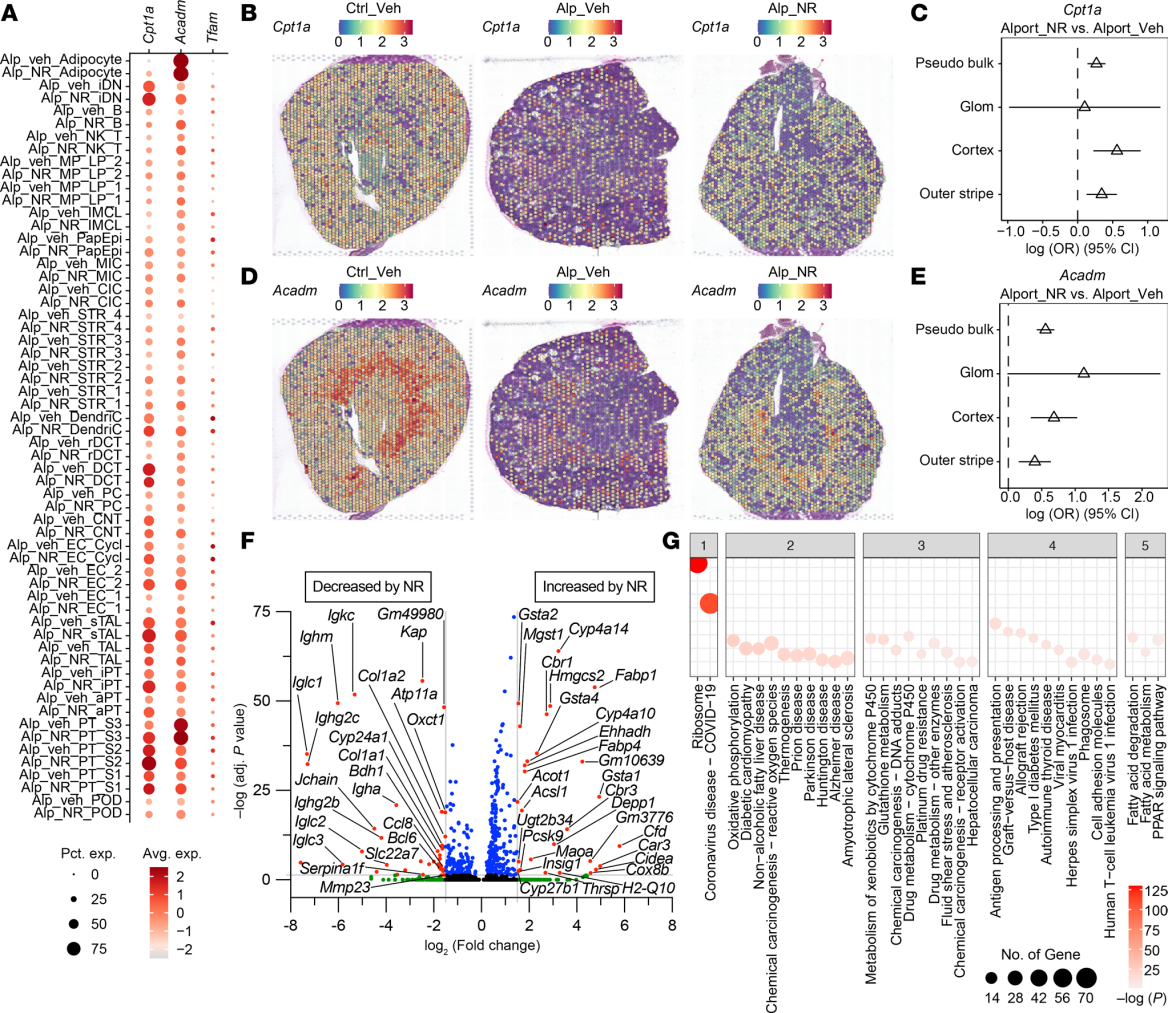
Spatial localization of *Cpt1a* and *Acadm*. (**A**) Expression of mitochondrial transcription factor A (*Tfam*), carnitine palmitoyltransferase 1-α (*Cpt1a*), and medium-chain acyl-coenzyme A dehydrogenase (MCAD; *Acadm*) in cell types and conditions by snRNA-Seq. (**B**) Visium spatial transcriptomics was performed on vehicle-treated male control mice (left), vehicle-treated male Alport mice (center), and NR-treated male Alport mice (right). *Cpt1a* expression is depicted. (**C**) *Cpt1a* expression was restored with NR treatment. (**D** and **E**) Expression of *Acadm* was reduced in Alport mice and restored after NR treatment. (**F**) Differentially expressed genes within the cortical tubulointerstitium in NR-treated Alport mice (vs. vehicle-treated Alport mice). (**G**) Pathway enrichment in the cortical tubulointerstitium in NR-treated Alport mice (vs. vehicle-treated Alport mice). Avg., average; CI, confidence interval; Cortex, cortical tubulointerstitium; Ctrl, control; Glom, glomerulus; NAD^+^, nicotinamide adenine dinucleotide; NR, nicotinamide riboside; OR, odds ratio with 95% CI; Pct., percentage; Veh, vehicle. aPT, adaptive proximal tubules; B, B cell; CIC, cortical intercalated cells; CNT, connecting tubule; DCT, distal convoluted tubule; DendriC, dendritic cell; EC_1, endothelial cells 1; EC_2, endothelial cells 2; EC_Cycl, endothelial cells cycling; iDN, injury distal nephron; IMCL, intramedullary collecting cells; iPT, injury proximal tubules; MIC, medullary intercalated cells; MP_LP_1, macrophage or lymphocyte cells 1; MP_LP_2, macrophage or lymphocyte cells 2; NK_T, natural killer or T cell; PapEpi, papillary epithelial cells; PC, principal cell; POD, podocyte; PT_S1, proximal tubules segment 1; PT_S2, proximal tubules segment 2 cells; PT_S3, proximal tubules segment 3 cells 1; rDCT, regenerative distal convoluted tubule; sTAL, stressed thick ascending limb; STR_1, stromal cell 1; STR_2, stromal cell 2; STR_3, stromal cell 3; STR_4, stromal cell 4; TAL, thick ascending limb.

We next sought to understand the cell type–specific and spatially anchored gene expression changes of *Cpt1a* and *Acadm* (MCAD) (Figure 8). *Cpt1a* and *Acadm* expression by snRNA-Seq was significantly increased in the proximal tubule S1 and S2 cells of Alport mice after NR treatment (Figure 8A). ST profiling supported the observed effects seen in the snRNA-Seq atlas. ST revealed that expression of *Cpt1a*, the rate-limiting step for mitochondrial long-chain FAO, was reduced in Alport mice and restored with NR treatment (Figure 8B and Supplemental Figure 31A). We assessed the spatial distribution of *Cpt1a* expression across all spots (pseudobulk) and in functional tissue units of glomeruli, the cortical tubulointerstitium (sans glomeruli), and the medullary outer stripe. These functional tissue units were selected by a combination of histologic assessment and marker gene expression with *Wt1* for glomeruli, *Slc34a1* for cortical tubulointerstitium, and *Slc3a1* for outer stripe (Supplemental Figure 32). *Cpt1a* expression was significantly upregulated in the pseudobulk, cortex, and outer stripe in Alport mice after NR treatment (Figure 8C). Similarly, *Acadm* expression was reduced in the Alport condition, and this reduction was mitigated with NR treatment (Figure 8, D and E, and Supplemental Figure 31B). The expression of *Cpt1a* and *Acadm* in glomeruli of Alport mice treated with NR trended toward an increase, though the number of glomerular spots was small. *Tfam* expression was not differentially expressed in the proximal tubule S1 or S2 cells of the snRNA-Seq dataset (Figure 8A and Supplemental Figure 31C). We assessed differential gene expression in the cortex of the ST samples (Figure 8F) and found similar pathways were enriched (Figure 8G) to those observed in the snRNA-Seq dataset, including the PPAR signaling pathway and fatty acid degradation.

Within the snRNA-Seq atlas, the proportions of myofibroblasts, macrophages, T lymphocytes, and B lymphocytes were all increased in Alport mice and reduced with NR treatment (Figure 7B). Using ST, these cell types were localized to the tissue with a label transfer method (Supplemental Figure 33). NR treatment led to reduced immune cell infiltration and stromal cell appearance in Alport mice.

Taken together, these data suggest that NR treatment restores the canonical functions of the proximal tubule, restores expression of *Cpt1a* and *Acadm*, reduces immune cell infiltration, and reduces myofibroblast appearance and fibrosis.

## Discussion

Herein, we have comprehensively shown that NR protects the kidney in a mouse model of Alport syndrome. In addition, we report several other noteworthy contributions. We believe we are the first to report single-cell (nuclei) and spatial transcriptomic data from the Alport model. We believe we are also the first to report sex differences in the bulk renal transcriptome of Alport mice. Finally, at both the bulk and single-cell (nuclei) levels, we report that NR-mediated renal activation of metabolism occurs in healthy control mice and diseased Alport mice. The similarity in response between both control and Alport mice strongly suggests that normalization of renal metabolism is responsible for the nephroprotective effect of NAD^+^ supplementation in CKD, a key mechanistic insight.

In our experiments, we identified that activation of renal metabolism in the proximal tubule via the NAD^+^/PGC-1α/PPARα/FAO axis is an important mechanism contributing to the nephroprotective effects of NAD^+^ supplementation in CKD. Our study builds upon prior research on the protective PPARα/FAO axis in Alport mice by identifying the importance of the NAD^+^/PGC-1α axis upstream of it (21). However, perhaps more importantly, we showed that activation of renal metabolism is, at least in part, the direct result of NAD^+^ supplementation — it is not just secondary to the prevention of kidney disease. The highly similar transcriptional responses of control and Alport mice to NAD^+^ supplementation allows us to make this key mechanistic insight, and it underscores the importance of also investigating the effects of drugs in the absence of disease. Nevertheless, although our results show that NAD^+^ supplementation activates renal metabolism and protects against CKD, they do not definitively demonstrate the role of NAD^+^ depletion in the pathogenesis of CKD. For that, further study using genetic knockout models would be needed.

Sex as a biological variable has been, and continues to be, a neglected area of research. It has been reported that male and female *Col4a3*^tm1Dec^ mice on the 129/SvJ background have similar severities of kidney disease (47). We note that this is unusual because male mice are generally more susceptible to kidney disease than female mice (48). Even though the current study was not designed as a comprehensive comparison between the sexes, we still identified moderate sex-specific differential regulation of fibrosis- and inflammation-related pathways at the transcriptional level, albeit not at the histological level. Our gene set represents a starting point that may assist in developing hypotheses to investigate a potential phenotypic sex difference in this mouse model of Alport syndrome.

Modern transcriptomic approaches, such as snRNA-Seq and ST, provide unparalleled insights into the molecular mechanisms of disease, and there is a focused effort to develop atlases with single-cell resolution (49). Because snRNA-Seq is still relatively new, datasets are not yet available for many disease models, especially rare diseases such as Alport syndrome. We believe our snRNA-Seq and ST datasets are the first of their kind from a model of Alport syndrome. In addition, we also report the first characterization to our knowledge of how NAD^+^ supplementation affects the kidney at the single-cell level in states of both health and disease. Our snRNA-Seq dataset is further differentiated by the inclusion of both sexes and the simultaneous assay for transposase-accessible chromatin, known as snATAC-Seq. However, a full comparison between the sexes, as well as integration of the multiome data, is beyond the scope of the current publication.

Although it has been known that exogenous NAD^+^ supplementation protects from CKD (11–16), the cell types that contribute to this benefit were not rigorously investigated. Models of AKI displayed the strongest and most consistent therapeutic benefit of NAD^+^ supplementation, implying the proximal tubules as a likely location. However, the data from models of CKD were less clear, and several studies suggested a potential role for podocytes (13, 50). Our data demonstrate the prominent role of the proximal tubule in NAD^+^-mediated protection from CKD. However, one limitation of our data was the relatively lower sample size of podocytes compared with proximal tubular epithelial cells in both the snRNA-Seq and ST datasets — a challenge common to many single-cell studies. While the effects of NAD^+^ supplementation were clear in the proximal tubule, whether NAD^+^ supplementation holds a direct effect on podocytes in Alport syndrome remains an outstanding question. The snRNA-Seq and ST data both suggest a trend toward restored *Cpt1a* and *Acadm* expression in most cell types and functional tissue units.

In summary, NAD^+^ supplementation protects the kidney in a mouse model of Alport syndrome. Mechanistically, NR activates renal metabolism by restoring the NAD^+^/PGC-1α/PPARα/FAO axis within the proximal tubules. Future directions include determining if NAD^+^ supplementation exerts similar effects on other cell types within the kidney and using genetic knockout models to definitively demonstrate the role of tubular NAD^+^ depletion in this model of CKD.

## Methods

### Sex as a biological variable.

All experiments were conducted in both sexes until we established a solid rationale for studying only a single sex. This rationale is comprehensively addressed in the Results.

### Animal models.

*Col4a3*^tm1Dec^ mice on the C57BL/6J background slowly develop kidney disease, and they were obtained as a gift from Sanofi (51, 52). Col4a3^–/–^ was the disease genotype, and Col4a3^+/–^ was the control genotype. Genotyping was performed by Transnetyx using reverse transcription quantitative PCR.

For all studies, mice were housed at ambient temperature with a 12-hour light/12-hour dark cycle and free access to food and water. Litter-to-litter variation was controlled by balancing treatment groups across litters. NR (CAS No. 1341-23-7) was obtained from ChromaDex through participation in its External Research Program. Heparinized plasma and organs were collected upon euthanasia with carbon dioxide.

### NAD^+^ quantification in control and Alport mice.

Control and Alport mice were maintained on a grain-based chow (catalog 3005740-220, LabDiet) and euthanized at 25 weeks old.

### NAD^+^ supplementation experiment in control and Alport mice.

Control and Alport mice were maintained on a grain-based chow until 10 weeks old. They were then switched to a purified control diet alone (catalog TD.130352, Envigo) or admixed with NR (0.5% w/w, catalog TD.190868, Envigo) for the remainder of the study. Administration of this dose of NR to mice via the diet has been reported previously (15). Photoplethysmography, echocardiography, and 24-hour urine collection were performed at 24–25 weeks old. Mice were euthanized at 25 weeks old.

### Replication of NAD^+^ supplementation experiment in Alport mice.

Control and Alport mice were maintained on a grain-based chow. NR was administered in the drinking water (5 g/L) to half of the mice, starting at 6 weeks of age. NR water was replaced twice per week. NR is stable in water dispensers for at least 6 days, and it does not affect water intake (53). Twenty-four–hour urine collection was performed at 24–25 weeks old, and mice were euthanized at 35 weeks old.

### In vivo measurements.

Urine was collected from mice by housing them in metabolic cages. Mice were habituated to the cages for 1 day prior to urine collection. Echocardiography (Vevo 3100, VisualSonics) was performed by an experienced preclinical ultrasound technician as previously described (54) and in accordance with recent guidelines (55). Blood pressure was measured by tail photoplethysmography (Model BP-2000-M-6, Visitech Systems). Habituation cycles were performed for the 2 days prior to collection of the blood pressure data.

### Biochemical assays.

NAD^+^ was quantified in a midtransverse kidney piece (catalog E2ND-100, BioAssay Systems). Urine albumin (catalog 1011, Ethos Biosciences) and plasma creatinine (catalog DICT-500, BioAssay Systems) were determined according to the manufacturers’ instructions. Expression of proteins of interest was quantified from a midtransverse kidney piece by immunoblotting and normalized to total protein using Ponceau S as previously described (22). The primary antibodies used for immunoblotting were CPT1α (catalog ab128568, Abcam), fibronectin (catalog F3648, MilliporeSigma), KIM-1 (catalog AF1817, R&D Systems, Bio-Techne), MCAD (catalog sc-49046, Santa Cruz Biotechnology), and PGC-1α (catalog AB3242, MilliporeSigma). The secondary antibodies were anti-rabbit (catalog A16110, Invitrogen), anti-goat (catalog sc-2768, Santa Cruz Biotechnology), and light chain–specific anti-mouse (catalog AP200P, MilliporeSigma).

### Histopathology and immunohistochemistry.

Tissues were drop-fixed in 10% neutral buffered formalin for 24 hours at 4°C, dehydrated with an ethanol-xylene gradient, and embedded in paraffin. FFPE tissues were sectioned (3 μm) onto glass slides using a microtome. PSR staining was performed (catalog SO-674, Rowley Biochemical), and polarized images were analyzed by thresholding in ImageJ (Fiji) (56). Renal cortical tubulointerstitial fibrosis was quantified using unpolarized PSR images after excluding glomerular, vascular, and medullary contributions in QuPath (57). PSR data are presented as the percentage of pixels that stained positive. Immunohistochemistry for p57^kip2^ (catalog ab75975, Abcam) followed by periodic acid–Schiff (catalog 22-110-645, Thermo Fisher Scientific) poststaining without hematoxylin counterstaining was performed and analyzed as previously described (26). Mesangial index was quantified using QuPath (57). Immunohistochemistry for CD45 (catalog 65087-1-Ig, Proteintech) was performed as previously described (22). Polarized images were acquired with an IX83 Inverted Microscope (Olympus Scientific Solutions). Unpolarized brightfield images were acquired with an Aperio GT 450 (Leica Microsystems) and a NanoZoomer (Hamamatsu Photonics).

### Analysis of previously published RNA-Seq datasets.

RNA-Seq data from previously published RNA-Seq experiments were downloaded from the NCBI Sequence Read Archive (SRA) (58). Accession numbers SRR1611815, SRR1611816, SRR1611817, SRR1611818, SRR1611819, SRR1611820, and SRR1611821 correspond to 3 male control and 4 male Alport kidneys, respectively, on the 129X1/SvJ background, at 5.5 weeks of age (21). Accession numbers SRR1611806, SRR1611807, SRR1611808, SRR1611809, SRR1611810, and SRR1611811 correspond to 3 male control and 3 male Alport kidneys, respectively, on the 129X1/SvJ background, at 9 weeks of age (21). Accession numbers SRR15102716, SRR15102717, SRR15102718, SRR15102719, SRR15102720, SRR15102721, SRR15102722, SRR15102723, SRR15102724, SRR15102725, and SRR15102726 correspond to 5 male control and 6 male Alport kidneys, respectively, on an F1 mixed 129/SvJ and C57BL/6J background, at 15 weeks of age (20). Sequencing files were aligned and processed with BioJupies, a Web server that automatically analyzes RNA-Seq datasets and generates Jupyter Notebooks (59). GO enrichment analyses and KEGG pathway analyses obtained from BioJupies were plotted in GraphPad Prism (31, 32).

### Bulk RNA-Seq and data analyses.

Bulk RNA-Seq was performed on isolated kidney cortex from 25-week-old *Col4a3*^tm1Dec^ mice on the C57BL/6J background. All 8 combinations of experimental groups were investigated: male vs. female, control vs. Alport, and vehicle vs. NR (*N* = 4 mice per group, 32 mice total). Total RNA was extracted with spin columns (catalog 74104, QIAGEN), and RNA-Seq using the poly(A) selection method was performed by GENEWIZ. Sequencing files were aligned, processes, and analyzed with BioJupies (59). GO enrichment analyses, KEGG pathway analyses, and transcription factor enrichment analyses (31–33) on the top 500 upregulated and downregulated genes were downloaded from BioJupies and further processed to identify statistically significant changes occurring in a priori–defined comparisons, as described in the Results.

Genes contributing to pathways that were reduced in Alport mice and restored by NR treatment were visualized by plotting KEGG graphs of the top 500 downregulated genes in vehicle-treated Alport mice (compared with vehicle-treated control mice) and the top 500 upregulated genes in NR-treated Alport mice (compared with vehicle-treated Alport mice) (Supplemental Table 8). Genes contributing to pathways activated by NR treatment were visualized by plotting KEGG graphs of the top 500 upregulated genes in NR-treated control mice (compared with vehicle-treated control mice) and the top 500 upregulated genes in NR-treated Alport mice (compared with vehicle-treated Alport mice) (Supplemental Table 9). KEGG graphs were rendered by Pathview Web (60, 61). Each gene contributed only once, and genes (primarily pseudogenes) that did not convert to the Ensembl namespace with gConvert were not plotted (62).

A separate analysis was performed to investigate a potential sex-genotype interaction. The subset of 16 vehicle-treated mice, 4 from each sex/genotype combination, were compared in a 2-by-2 factorial design using iDEP, a web application for analyzing previously aligned RNA-Seq data (40). Differential gene expression between the sexes, the genotypes, and the interaction term was calculated. Genes with both at least a 2-fold change in expression and an FDR-adjusted *P* value less than 0.05 were deemed statistically significant. All principal component analyses were performed using iDEP and plotted with GraphPad Prism (40).

A separate analysis was performed to investigate a potential effect of mouse genetic background. The subset of 8 vehicle-treated male mice, 4 from each genotype, were compared with control and Alport mice on the 129 and mixed B6/129 backgrounds. A meta-analysis was performed using ExpressAnalyst as previously described (63, 64). Briefly, raw read counts were filtered, log_2_-normalized, and batch-corrected. *P* values from genes with FDR-adjusted *P* values less than 0.05 in at least 1 study were combined using Fisher’s method. KEGG pathway analyses were performed on genes with at least a 2-fold change in expression and an FDR-adjusted *P* value less than 0.05 (65).

### snRNA-Seq.

snRNA-Seq and simultaneous snATAC-Seq were performed on kidneys from 25-week-old *Col4a3*^tm1Dec^ mice on the C57BL/6J background. All 8 combinations of experimental groups were investigated: male vs. female, control vs. Alport, and vehicle vs. NR (*N* = 1 mouse per group, 8 mice total). Male and female nuclei of the same genotype/treatment combination were pooled, and only the pooled snRNA-Seq data are presented here.

For snRNA-Seq, 49,488 nuclei were isolated from kidney cryosections and processed using the Chromium Next GEM Single Cell Multiome ATAC + Gene Expression (v1.0) kit as previously described (66). The RNA and ATAC libraries were sequenced separately on an Illumina NovaSeq 6000 system (v.1.7.0 and v.1.7.5). For RNA analysis, cell barcodes passing the following quality control filters were used for downstream analyses: (a) passed 10x Genomics Cell Ranger Arc (RNA) filters, (b) nCount_RNA showing greater than 1,000 and fewer than 25,000 in nonmitochondrial genes detected, and (c) cells with percentage of mitochondrial transcripts greater than 25% were removed. RNA counts were normalized using sctransform, and the clustering was done with 0.5 resolution. This integration was performed using Seurat (v.5.0.0). Cluster annotations were based on published gene markers (67) and converted by BioMart (v.2.56.1) to the mouse homologous genes. The differential expressed genes are identified by RunPresto with logFC threshold = 0, Wilcox test, and corrected by Bonferroni from package SeuratWrappers (v.0.3.2). The pathway analyses were performed by pathfindR (v.2.3.0.9001) (68). The mmu_STRING database to identify relevant networks of protein interactions and mmu_KEGG database to contextualize these networks within known biological pathways are enriched.

### ST.

ST was performed on kidneys from 25-week-old *Col4a3*^tm1Dec^ mice on the C57BL/6J background. All 4 combinations of male experimental groups were investigated: control versus Alport and vehicle versus NR (*N* = 1 male mouse per group, 4 mice total). The same tissue blocks were used for both snRNA-Seq/snATAC-Seq and ST.

Murine kidney tissues were embedded in O.C.T. compound (catalog 23-730-571, Thermo Fisher Scientific) immediately after euthanasia. Samples were processed according to the Visium Spatial Gene Expression protocol (10x Genomics, CG000240 protocol) (69). One sample from each condition underwent cryosectioning to yield a section of 10 μm thickness. The sections were stained with periodic acid–Schiff and imaged using a Keyence BZ-X810 microscope, equipped with a Nikon 10× CFI Plan Fluor objective lens. The brightfield images were compiled and matched with Visium fiducials to create comprehensive mosaics. The mRNA from the tissue sections was extracted following a 12-minute permeabilization period. This mRNA then adhered to oligonucleotides at the fiducial spots and was subsequently reverse-transcribed. In the subsequent stages of library creation and sequencing, the mRNA was converted into second-strand cDNA. This was followed by denaturation, amplification of the cDNA, and purification using SPRIselect cDNA cleanup (Visium CG000239 protocol). Finally, the cDNA sequencing was performed using an Illumina NovaSeq 6000 system. For spatial analysis, Space Ranger (v2.0.0) with the reference mouse genome (mm10-2020-A) was used to perform expression analysis, mapping, counting, and clustering. Labels were transferred from snRNA-Seq to ST to spatially localize the cell types based on gene expression profiles using Seurat (v.5.0.0) onto 8,748 spots.

In ST samples, spots with positive expression of *Nphs2* and *Wt1* were annotated as glomeruli. Conversely, spots were annotated as cortical proximal tubule when positive expression of *Slc34a1* was observed without expression of *Nphs2* and *Wt1*. The outer stripe of the medulla was selected by the presence of *Slc3a1* expression above *Slc34a1* expression and without *Nphs2* or *Wt1* expression. Each spot could be assigned to only a single functional tissue unit. An investigator confirmed annotations of each spot using histology or excluded spots if the histology was inconsistent with the marker gene expression. A pseudobulk comparison was made across all spots in the sample. Differential expression between the Alport NR and Alport Vehicle spots for each annotation was evaluated with a Mann-Whitney test. Pathway enrichment was performed with pathfinder (31608109) using STRING network and KEGG pathways. For *Cpt1a* and *Acadm*, spots were classified as nonzero if expression was higher than 0 or 0 if no expression was detected. An odds ratio with 95% confidence interval was used to assess the likelihood of nonzero spots localizing to each region.

### Statistics.

One-way ANOVA was performed with GraphPad Prism. For ANOVA post hoc tests, the decision to compare only the following groups was made a priori: (a) control + vehicle vs. control + NR, (b) control + vehicle vs. Alport + vehicle, (c) Alport + vehicle vs. Alport + NR, and (d) control + vehicle vs. Alport + NR. *P* < 0.05 was considered statistically significant.

### Study approval.

Animal studies were approved by the Institutional Animal Care and Use Committee of Georgetown University and adhered to standards set by the Public Health Service Policy on Humane Care and Use of Laboratory Animals (National Institutes of Health, 2024).

### Data availability.

The values for data points in graphs are reported in the Supporting Data Values. The raw data from the bulk RNA-Seq, snRNA-Seq, and ST experiments have been deposited in NCBI’s SRA (58) and are accessible through SRA BioProject accession number PRJNA1088395. The processed transcriptomic data discussed in this publication have been deposited in NCBI’s GEO (70) and are accessible through GEO Series accession numbers GSE261869, GSE261871, and GSE261872.

## Author contributions

BAJ and ML conceived the study. BAJ and ML developed methodology. BAJ, DLG, RMF, BAS, and MTE developed software. BAJ, DLG, BAS, and ML performed validation. BAJ, DLG, ET, JP, RMF, and MTE performed formal analysis. BAJ, AS, YHC, KK, and XY performed investigation. BAJ, SJ, MTE, and ML provided resources. BAJ, DLG, RMF, and MTE curated data. BAJ, DLG, and MTE wrote the original draft. BAJ, DLG, KM, AS, YHC, ET, JP, KK, RMF, XY, BAS, KCA, TY, XXW, AZR, SJ, MTE, and ML reviewed and edited the manuscript. BAJ and DLG performed visualization. ML supervised and administered the project. BAJ, SJ, MTE, and ML acquired funding.

## Supplementary Material

Supplemental data

Unedited blot and gel images

Supplemental table 4

Supplemental table 5

Supplemental table 6

Supplemental table 7

Supplemental table 8

Supplemental table 9

Supporting data values

## Version 1 03/10/2025

Electronic publication
