# Supplementary material for: NAD+ prevents chronic kidney disease by activating renal tubular metabolism
Source: JCI Insight. 2025 Mar 10;10(5):e181443. doi: 10.1172/jci.insight.181443 (PMC11949063; doi:10.1172/jci.insight.181443)

NAD^+ prevents chronic kidney disease by activating renal tubular metabolism

Unedited Blot and Gel Images

Table of Contents

Immunoblot and Ponceau S for Figure 3E 2
Immunoblot and Ponceau S for Figure 3F 3
Immunoblot and Ponceau S for Figure 6A 4
Immunoblot and Ponceau S for Figure 6C 5
Immunoblot and Ponceau S for Figure 6D 6
Immunoblot and Ponceau S for Figure S4A 7
Immunoblot and Ponceau S for Figure S4B 8

Immunoblot and Ponceau S for Figure 3E

Kidney Injury Molecule-1 (KIM-1)

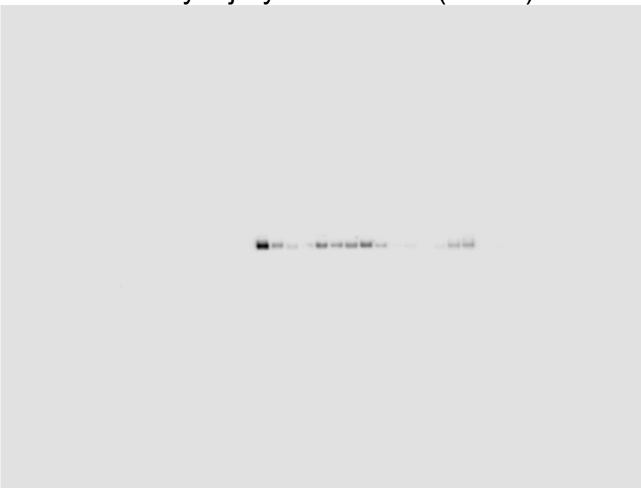

Total Protein

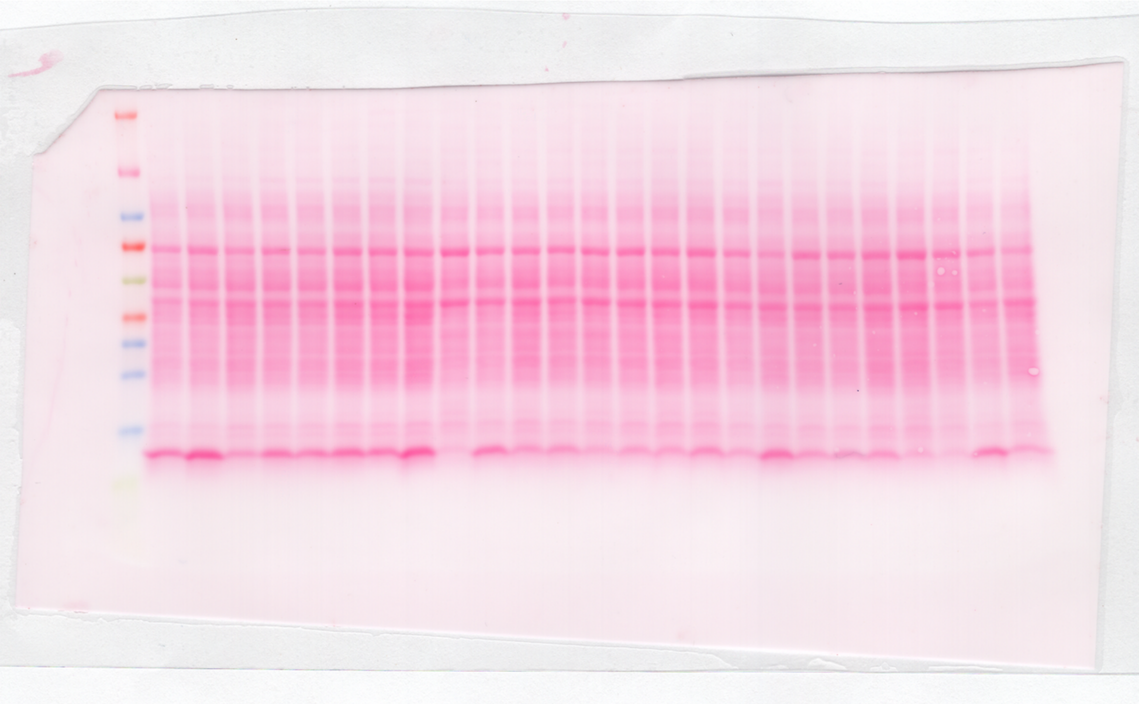

Immunoblot and Ponceau S for Figure 3F

Kidney Injury Molecule-1 (KIM-1)

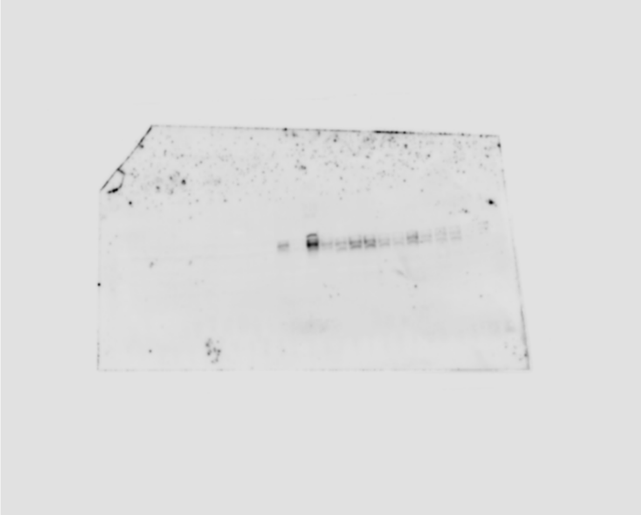

Total Protein

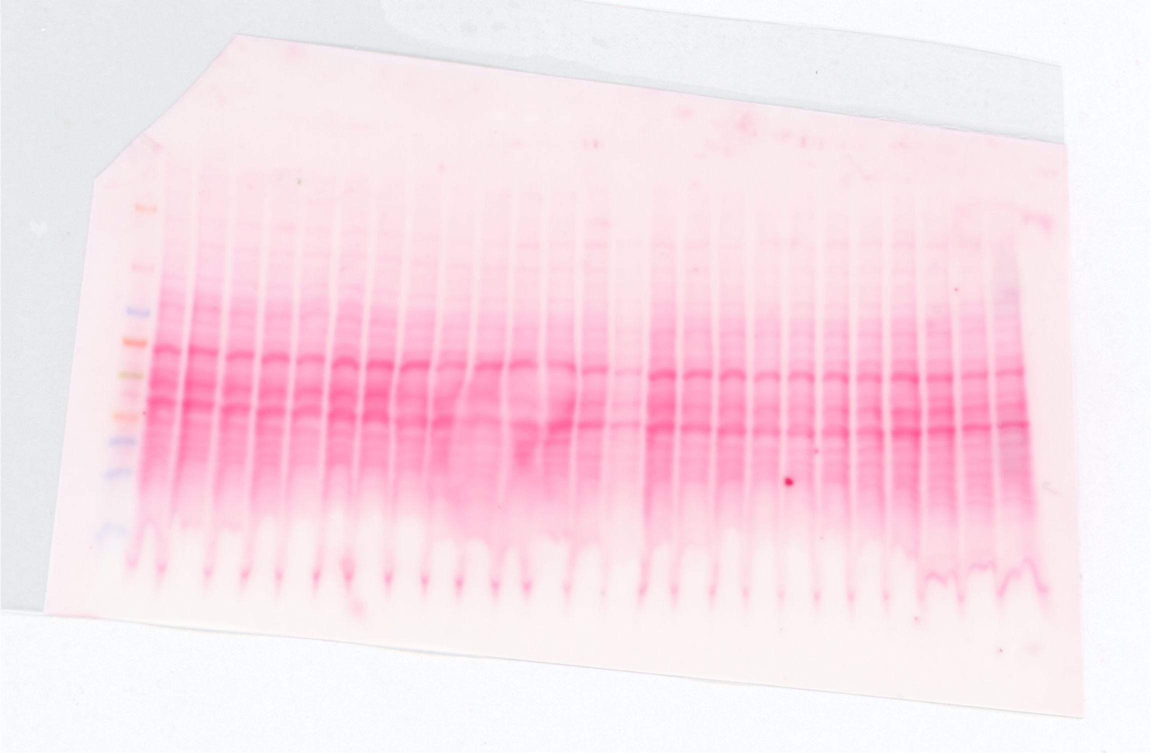

Immunoblot and Ponceau S for Figure 6A

Peroxisome proliferator-activated receptor gamma coactivator 1 alpha (PGC-1α)

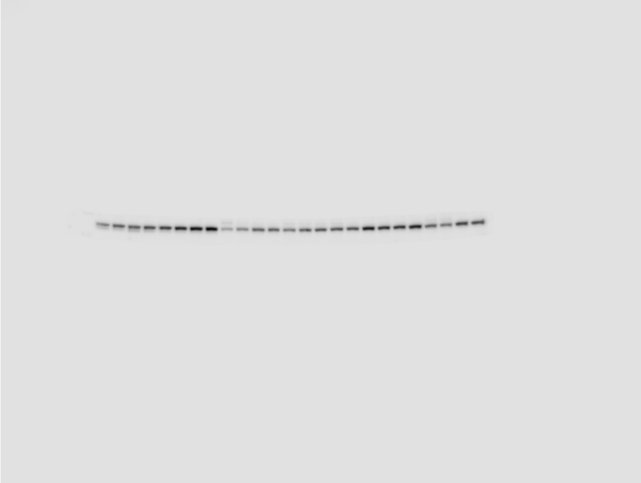

Total Protein

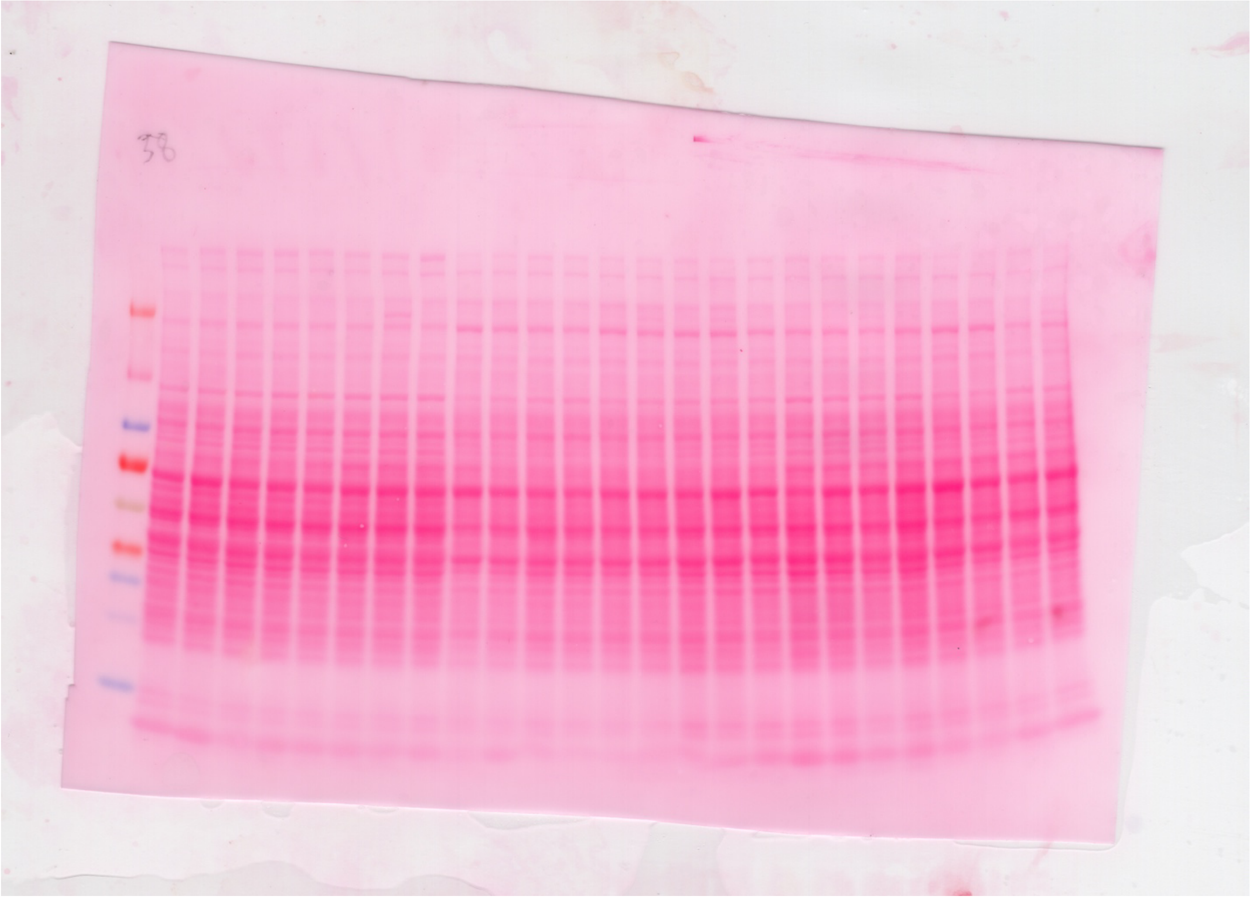

This immunoblot was horizontally cut prior to incubation with the primary antibodies for fibronectin (**Fig. S4A**) and PGC-1α (**Fig. 6A**). Therefore, data from those blots were normalized to the same Ponceau S image, presented above and on page 7.

Immunoblot and Ponceau S for Figure 6C

Carnitine palmitoyltransferase 1 alpha (CPT1α)

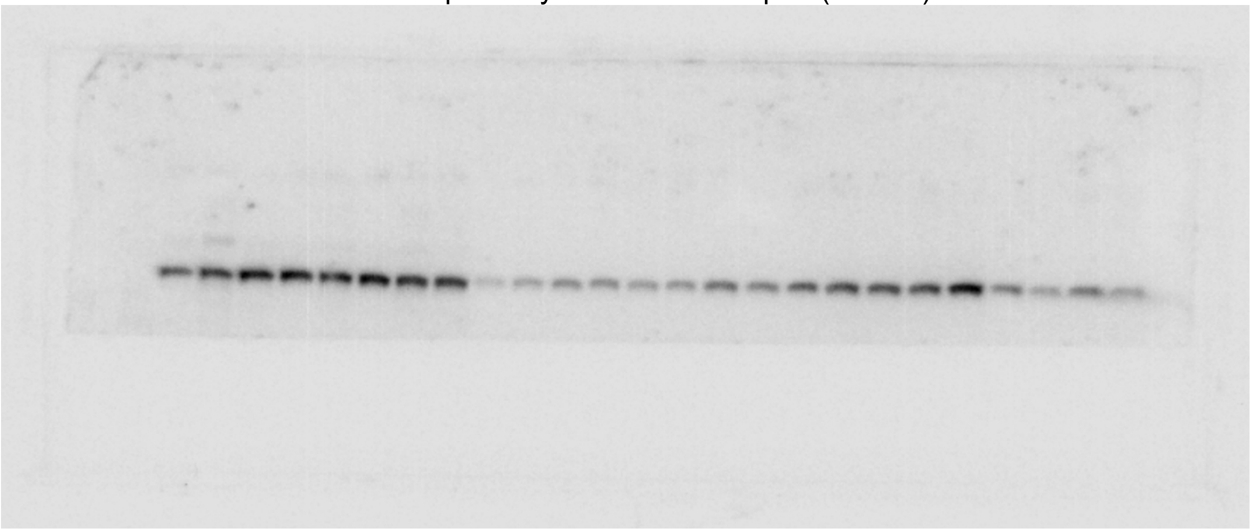

Total Protein

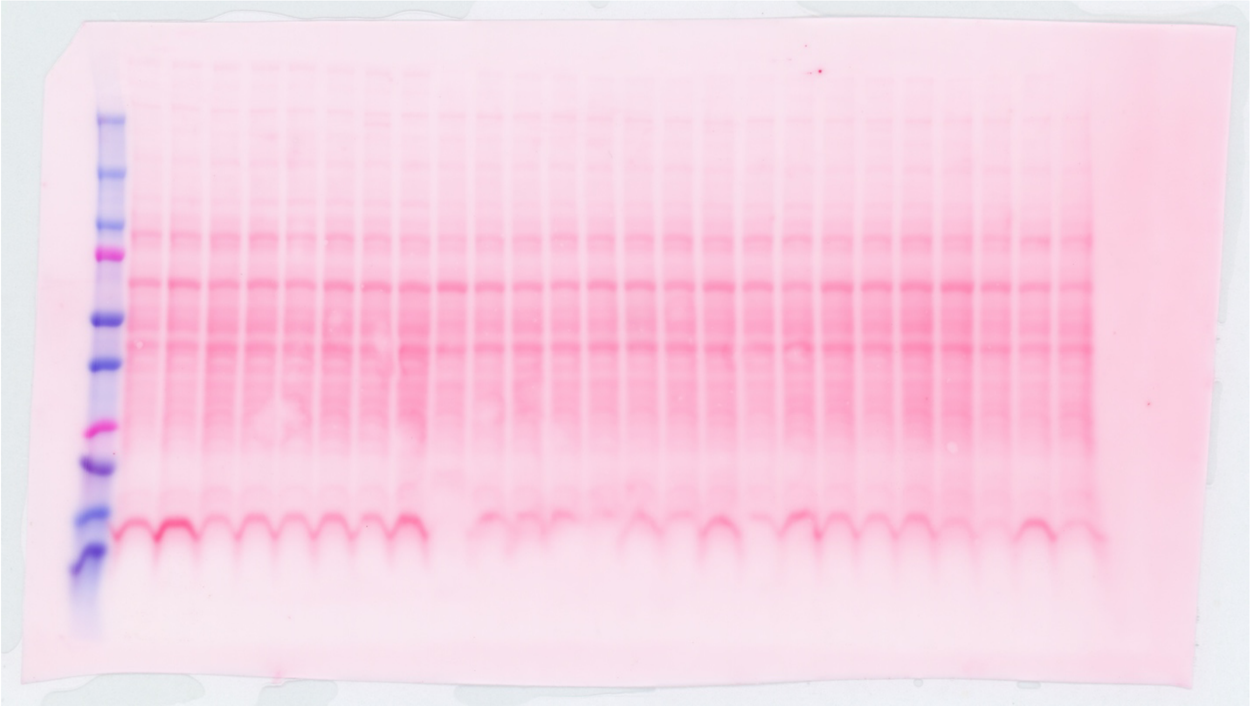

Immunoblot and Ponceau S for Figure 6D

Medium-chain acyl-coenzyme A dehydrogenase (MCAD)

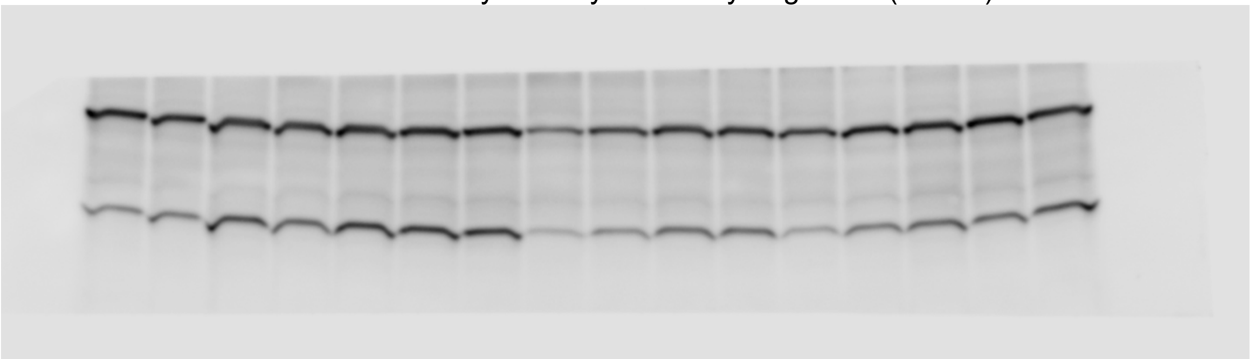

Total Protein

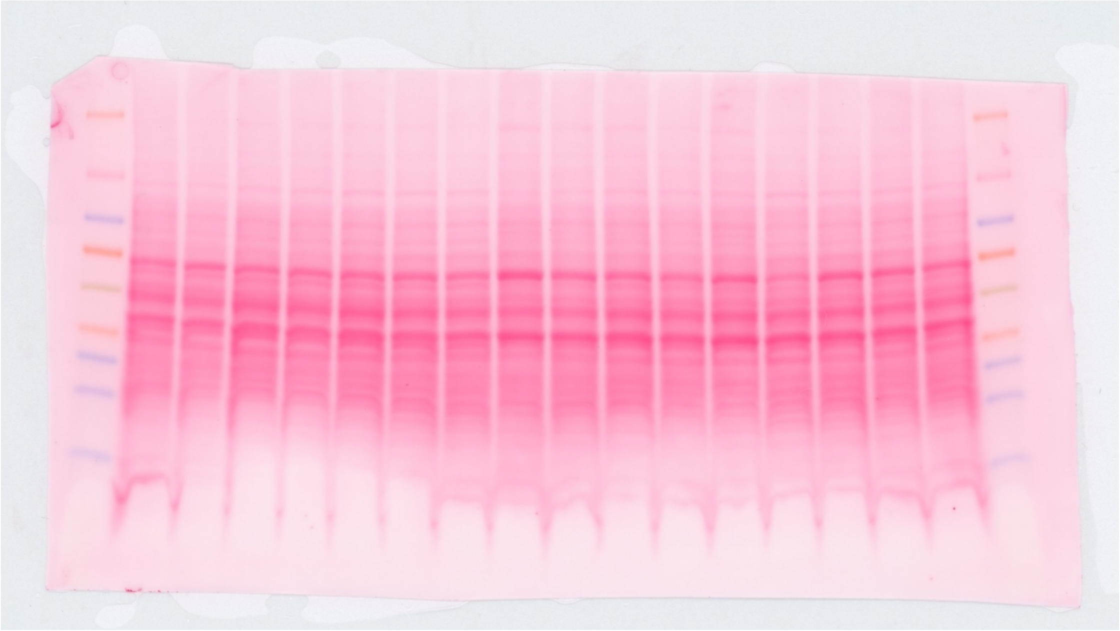

Immunoblot and Ponceau S for Figure S4A

Fibronectin

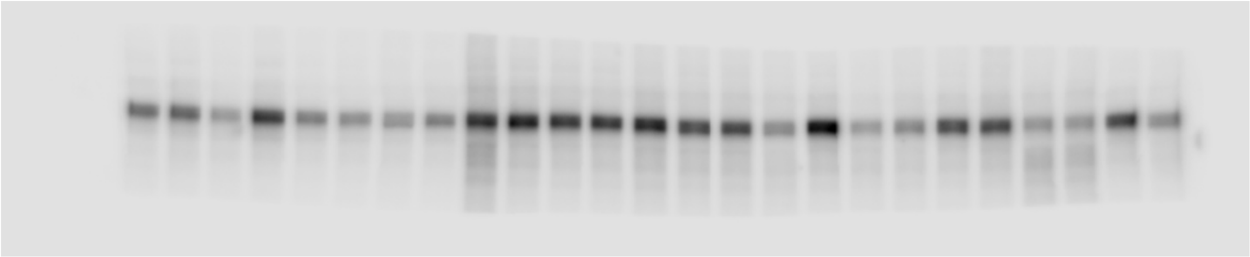

Total Protein

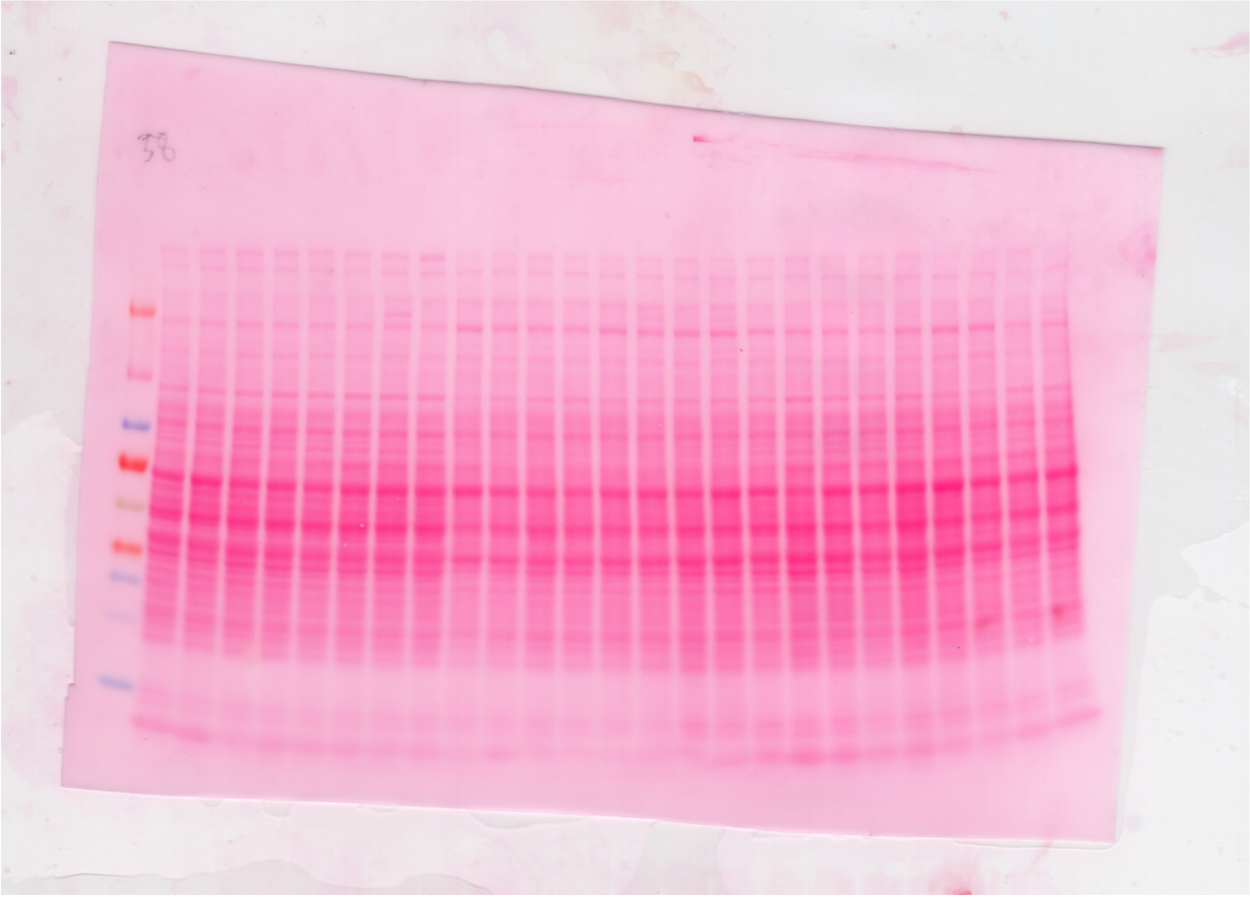

This immunoblot was horizontally cut prior to incubation with the primary antibodies for fibronectin (**Fig. S4A**) and PGC-1α (**Fig. 6A**). Therefore, data from those blots were normalized to the same Ponceau S image, presented above and on page 4.

Immunoblot and Ponceau S for Figure S4B

Fibronectin

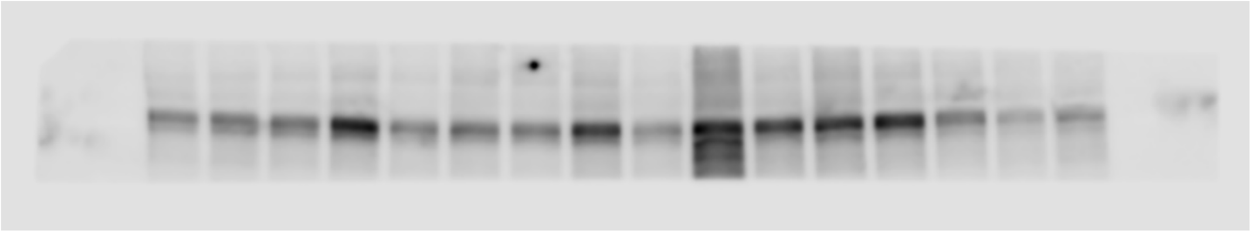

Total Protein

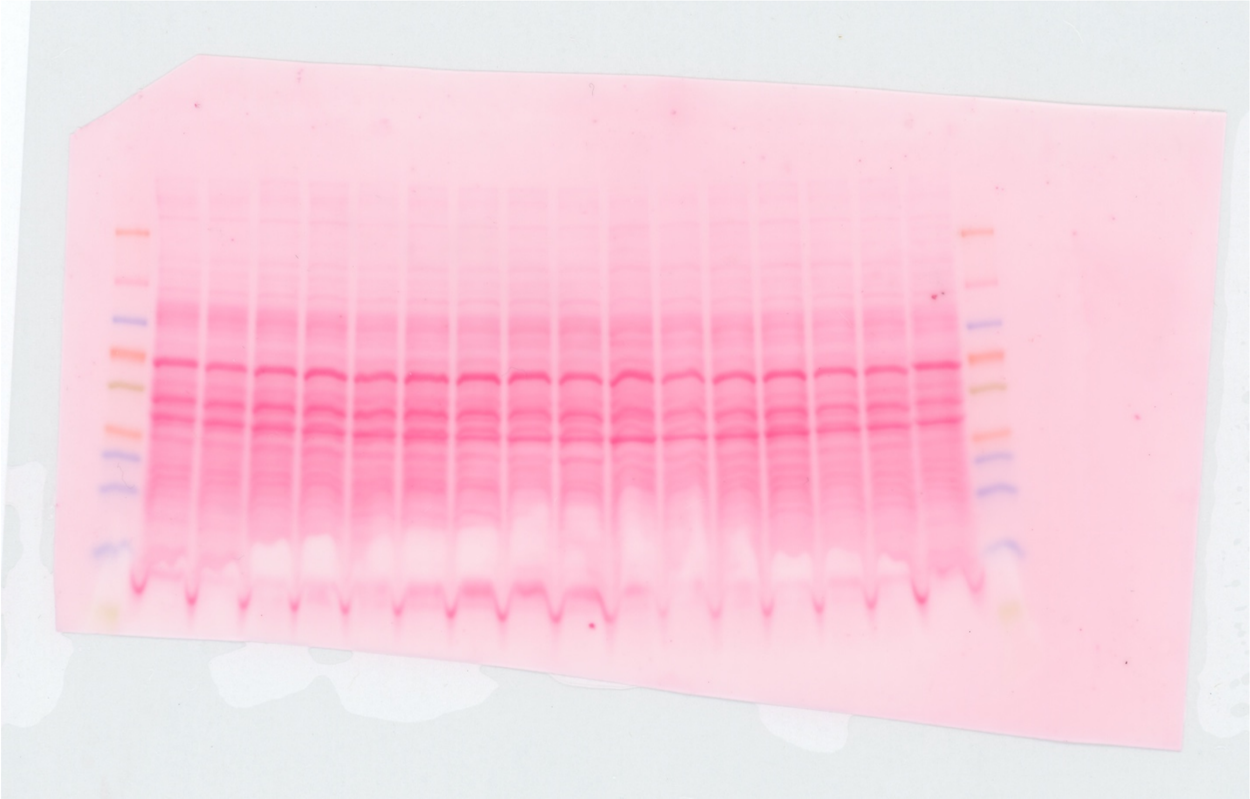

Supplement: Unedited blot and gel images [file jciinsight-10-181443-s066.pdf]
